# Expanding our Understanding of Sequence-Function Relationships of Type II Polyketide Biosynthetic Gene Clusters: Bioinformatics-Guided Identification of Frankiamicin A from *Frankia* sp. EAN1pec

**DOI:** 10.1371/journal.pone.0121505

**Published:** 2015-04-02

**Authors:** Yasushi Ogasawara, Benjamin J. Yackley, Jacob A. Greenberg, Snezna Rogelj, Charles E. Melançon

**Affiliations:** 1 Department of Chemistry and Chemical Biology, University of New Mexico, Albuquerque, New Mexico, United States of America; 2 Department of Biology, University of New Mexico, Albuquerque, New Mexico, United States of America; 3 Center for Biomedical Engineering, University of New Mexico, Albuquerque, New Mexico, United States of America; 4 Department of Chemistry, New Mexico Institute of Mining and Technology, Socorro, New Mexico, United States of America; 5 Department of Biology, New Mexico Institute of Mining and Technology, Socorro, New Mexico, United States of America

## Abstract

A large and rapidly increasing number of unstudied “orphan” natural product biosynthetic gene clusters are being uncovered in sequenced microbial genomes. An important goal of modern natural products research is to be able to accurately predict natural product structures and biosynthetic pathways from these gene cluster sequences. This requires both development of bioinformatic methods for global analysis of these gene clusters and experimental characterization of select products produced by gene clusters with divergent sequence characteristics. Here, we conduct global bioinformatic analysis of all available type II polyketide gene cluster sequences and identify a conserved set of gene clusters with unique ketosynthase α/β sequence characteristics in the genomes of *Frankia* species, a group of Actinobacteria with underexploited natural product biosynthetic potential. Through LC-MS profiling of extracts from several *Frankia* species grown under various conditions, we identified *Frankia* sp. EAN1pec as producing a compound with spectral characteristics consistent with the type II polyketide produced by this gene cluster. We isolated the compound, a pentangular polyketide which we named frankiamicin A, and elucidated its structure by NMR and labeled precursor feeding. We also propose biosynthetic and regulatory pathways for frankiamicin A based on comparative genomic analysis and literature precedent, and conduct bioactivity assays of the compound. Our findings provide new information linking this set of *Frankia* gene clusters with the compound they produce, and our approach has implications for accurate functional prediction of the many other type II polyketide clusters present in bacterial genomes.

## Introduction

**Fig 1:**
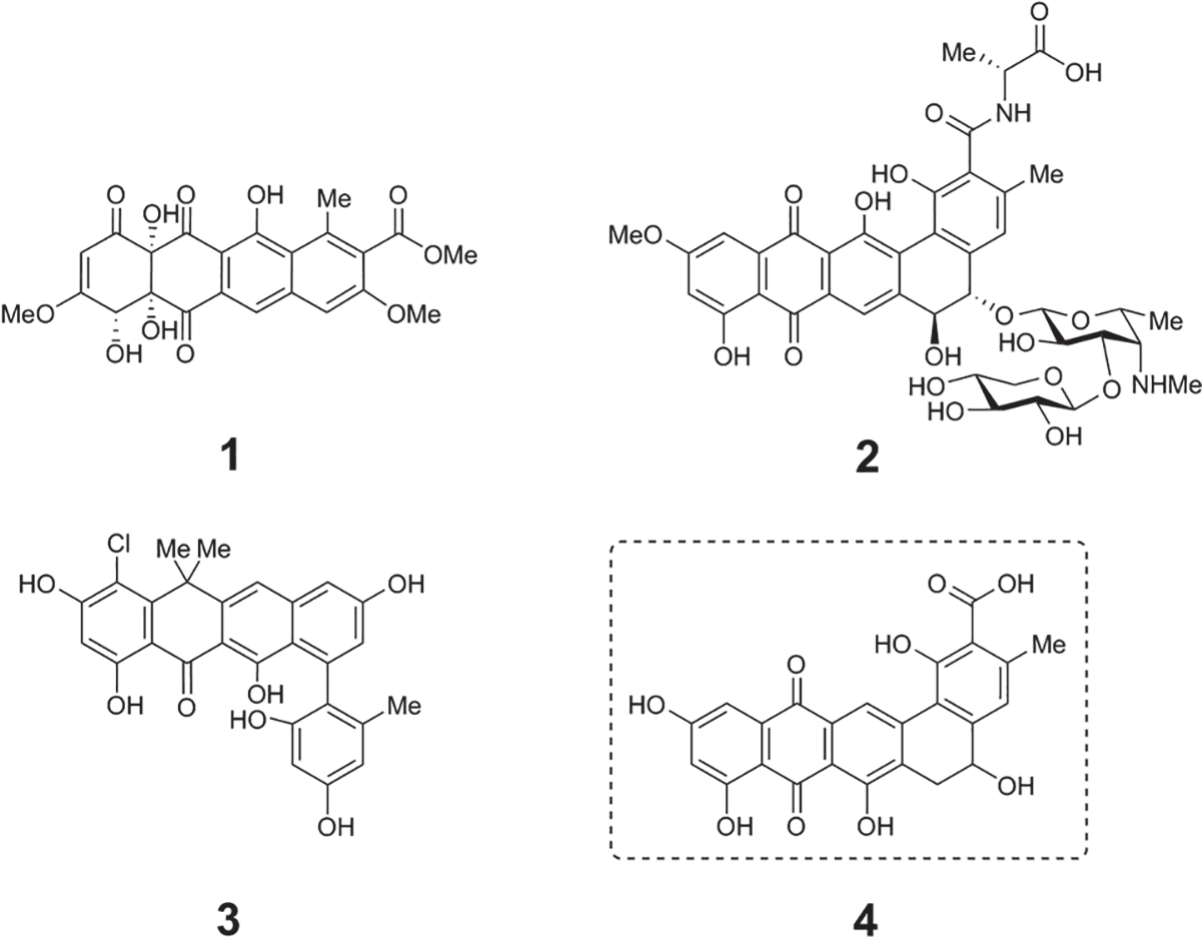
Structures of prototypical type II polyketides. Structures of chlortetracycline (**1**), doxorubicin (**2**), R1128A (**3**), and the pentangular polyketide frankiamicin A (**4**) identified in this study.

Polyketides are a structurally diverse family of natural products known for their medicinally useful bioactivities [1,2] as well as for their ecological roles [3]. Among these, members of the bacterial type II polyketide class, exemplified by the antitumor agent tetracenomycin C (**1**) [4], the antifungal pradimicin A (**2**) [5,6], and the antibacterial compound fasamycin A (**3**) [7] are characterized by planar aromatic fused ring core structures and a common biosynthetic origin (Fig. 1) [8].

**Fig 2:**
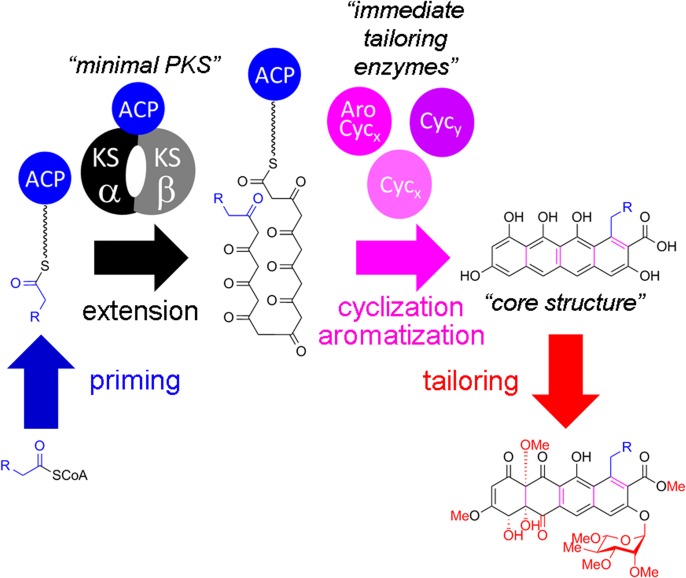
General summary of type II polyketide biosynthesis. The key steps in type II polyketide biosynthesis—priming of the minimal polyketide synthase, extension of the polyketide chain by the ketosynthase α/β heterodimer to generate the poly-β-ketone intermediate, cyclization and aromatization of the poly-β-ketone by the immediate tailoring enzymes (aromatase/cyclase and cyclases) to form the cyclized core structure, and tailoring by various polyketide tailoring enzymes—are shown, using the elloramycin biosynthetic pathway as an example. Structural elements of the intermediates and final product are color-coded according to which enzymes catalyze their formation.

In bacterial type II polyketide biosynthesis, the ketosynthase α/β/acyl carrier protein (KSα/β/ACP) “minimal polyketide synthase” complex [9] is responsible for iterative Claisen condensation of an ACP-bound starter unit and a specific number of malonyl-CoA-derived acetate extender units to generate a poly-β-ketone chain of defined length. These poly-β-ketone intermediates then undergo a series of regiospecific “immediate tailoring” reactions—optional C-9 ketoreduction, cyclizations, and aromatizations—to form planar aromatic “core structures”, the first stable pathway intermediates. These core structures are then elaborated by a myriad of tailoring enzymes, including oxygenases, methyltransferases, reductases, and glycosyltransferases (Fig. 2) [8].

The KSα/β heterodimer controls the chain length of the poly-β-ketone intermediate, with 16- to 30-carbon chains known thus far. The crystal structure of the actinorhodin KSα/β heterodimer [10] and the results of bioengineering studies [11] have led to the proposal that the size and shape of the KSα/β active site control the length of the poly-β-ketone produced. Cyclization and dehydration reactions are catalyzed by specific sets of three to four cyclases to form particular planar aromatic core structures characteristic of each type II polyketide structural subclass. Six fold families of cyclase, some comprised of two or more subfamilies, each with its own reaction specificity, are currently known [12]. Interestingly, aromatic polyketides with structural similarities to bacterial type II polyketides, but made by a different set of enzymatic machinery, are also found in fungi [13].

The rapidly increasing number of available genome sequences has revealed that the genetic capacity to produce natural products, including bacterial type II polyketides, is widespread, and extends to many bacterial genera that are unexploited or underexploited with respect to natural products. The existence of a vast untapped reservoir of natural product gene clusters in microbial genome sequences underscores the need for systematic, combined bioinformatic/experimental approaches to more completely understand natural product gene and gene cluster sequence/function relationships and to more efficiently link gene clusters with the compounds they produce. Application of such approaches will, over time, expand and organize the collective knowledge base on natural product biosynthesis, allowing increasingly rapid, accurate, and large-scale prediction, elucidation, and bioengineering of natural product pathways and compound structures from gene cluster sequences. Similar approaches have been successfully applied to studying sequence/function relationships in enzyme superfamilies [14] and for operons involved in primary metabolism in microbes [15].

Recently, bioinformatic analysis has begun to play an increasingly prominent role in natural product discovery and biosynthesis studies [16]. A number of bioinformatics software packages such as antiSMASH [17], NP.searcher [18], and CLUSEAN [19] have been developed to automatically identify, annotate, and classify natural product gene clusters and to predict product structures given user-input DNA or protein sequences. Such software packages greatly facilitate annotation of individual newly-sequenced gene clusters and identification and classification of gene clusters from whole genome sequencing projects. However, the limited ability of these software packages to perform database-wide comparative gene and gene cluster analyses limits their utility for systematic study of sequence/function relationships. For such studies it is desirable to be able to globally survey all natural product gene clusters representing a particular biosynthetic class and select for experimental characterization clusters that are representative of groups with unique gene sequence characteristics or unique gene compositions. Some currently available software packages are also unable to identify bacterial type II polyketide gene clusters, and none are able to predict which structural subclass a type II polyketide gene cluster produces. PKMiner, a database of 40 unstudied type II polyketide gene clusters from sequenced bacterial genomes, which includes structural subclass predictions, was recently reported [20]. However, the PKMiner database must be manually updated, is incomplete, and lacks the necessary features to conduct global comparative analysis of bacterial type II polyketide genes and gene clusters.

Here we report use of our natural product bioinformatics software package *Dynamite*, which has unique capabilities beyond those of currently available software packages that facilitate global comparative analysis of natural product gene clusters (see Materials and Methods section, bioinformatic analysis subsection for details), together with metadata on poly-β-ketone structures and structural subclasses mined from literature on a training set of 64 studied bacterial type II polyketide clusters, to globally identify and annotate all bacterial type II polyketide gene clusters present in the NCBI databank and to provide predictive information on compound structures produced by these clusters. To correlate training set ketosynthase α/β (KSα/β) sequences with poly-β-ketone chain lengths and to explore the possibility of predicting poly-β-ketone structures from KSα/β sequences, we carried out dendrogramatic analysis of all ketosynthase α/β (KSα/β) sequences within these gene clusters. This analysis revealed strong correlations between the positions of KSα/β sequences in the dendrogram and both poly-β-ketone structure and structural subclass for training set members.

Interestingly, KSα/β dendrogramatic analysis revealed a clade of KSα/β sequences found exclusively in unstudied gene clusters, most of which occur in the genomes of *Frankia* species, whose sequences were sufficiently diverged from studied systems that the product poly-β-ketone chain lengths could not be predicted. Further comparative analysis of remaining biosynthetic genes in the *Frankia* clusters revealed strong gene synteny among the clusters and high similarity of encoded proteins to immediate tailoring enzymes involved in biosynthesis of type II polyketides from the pentangular and tetracenomycin subclasses.

To determine the polyketide chain length produced by this KSα/β clade and the structure of the product made by these gene clusters, we screened extracts from three *Frankia* species harboring the cluster to identify and structurally characterize the compound. Among the three strains, we identified *Frankia* sp. EAN1pec alone as producing a compound with spectral characteristics consistent with those of the predicted type II polyketide. Isolation and structure elucidation of the compound revealed it to be the pentangular type II polyketide **4**, which we named frankiamicin A (Fig. 1), thereby revealing that the KSα/β is a member of a new 24 carbon poly-β-ketone-producing clade. Furthermore, we use comparative genomics to propose a refined model for the functions of biosynthetic enzymes encoded in the frankiamicin cluster and other pentangular clusters; and we conduct initial bioactivity studies of the compound.

## Materials and Methods

### General

All chemicals including media components were purchased from Sigma-Aldrich (St. Louis, MO), VWR (Radnor, PA) or Fisher Scientific (Pittsburgh, PA) and were used without further purification. HPLC analysis was performed using a Dionex Ultimate 3000 instrument equipped with a photo diode array (PDA) detector and the specified column (see below). LC-MS analysis was performed using an API 2000 electrospray ionization (ESI) mass spectrometer (AB SCIEX) connected to the HPLC system. Post-column splitting (1:4) was used to simultaneously monitor MS and uv-visible spectra. NMR spectra were obtained using Bruker Avance III 300 and Avance 500 spectrometers housed in the NMR Core Facility in the Department of Chemistry and Chemical Biology at the University of New Mexico. Chemical shifts (δ in parts per million) are reported relative to that of the solvent peak (δ = 2.50 ppm and 39.5 ppm for DMSO-*d*_6_ in ^1^H and ^13^C NMR spectra, respectively). High resolution MS data was obtained using a Waters LCT Premier ESI-TOF mass spectrometer housed in the Mass Spectrometry and Proteomics Core Facility in the Department of Chemistry and Chemical Biology at the University of New Mexico. Vector NTI Advance 10 (Life Technologies, Carlsbad, CA) was used for routine sequence analysis.

### Bioinformatic analysis

The Python-based software package *Dynamite*, which we are currently developing, was used to identify natural product biosynthetic gene clusters encoded in nucleotide/protein sequences within in the entire NCBI databank. The *Dynamite* automated workflow is as follows (see S1 Fig.): 163 protein sequences representing many conserved protein families found in type I and type II polyketide and non-ribosomal peptide gene clusters are used to query a locally-housed NCBI protein databank using the blastp algorithm [21]. Hits and associated metadata (including species, GI number, and other attributes) obtained using these queries are sorted based on GI number, which arranges them according to their positions within genomes, identifying putative natural product gene clusters. Gene clusters are then classified by biosynthetic characteristics (type I polyketide synthase, type II polyketide synthase, non-ribosomal peptide synthetase) based on the presence of specific sets of hits within a particular GI number range. Summaries of the attributes (species, GI number range, arrangement of hit types from each gene cluster on the genome, biosynthetic classification) of all gene clusters found, as well as of gene clusters that conform to specific biosynthetic classifications, are output as text files that can be viewed and analyzed by the user. All protein sequences corresponding to specific hit types (e.g. KSα, KSβ) from specific gene cluster biosynthetic types can also be compiled in a semi-automated manner using a script within *Dynamite*, and output as multi-fasta files for further analysis. Gene clusters displaying biosynthetic characteristics of interest can also be manually downloaded as. gb files from NCBI, guided by *Dynamite* summary files, and subjected to further manual analysis using standard software such as Vector NTI.

**Fig 3:**
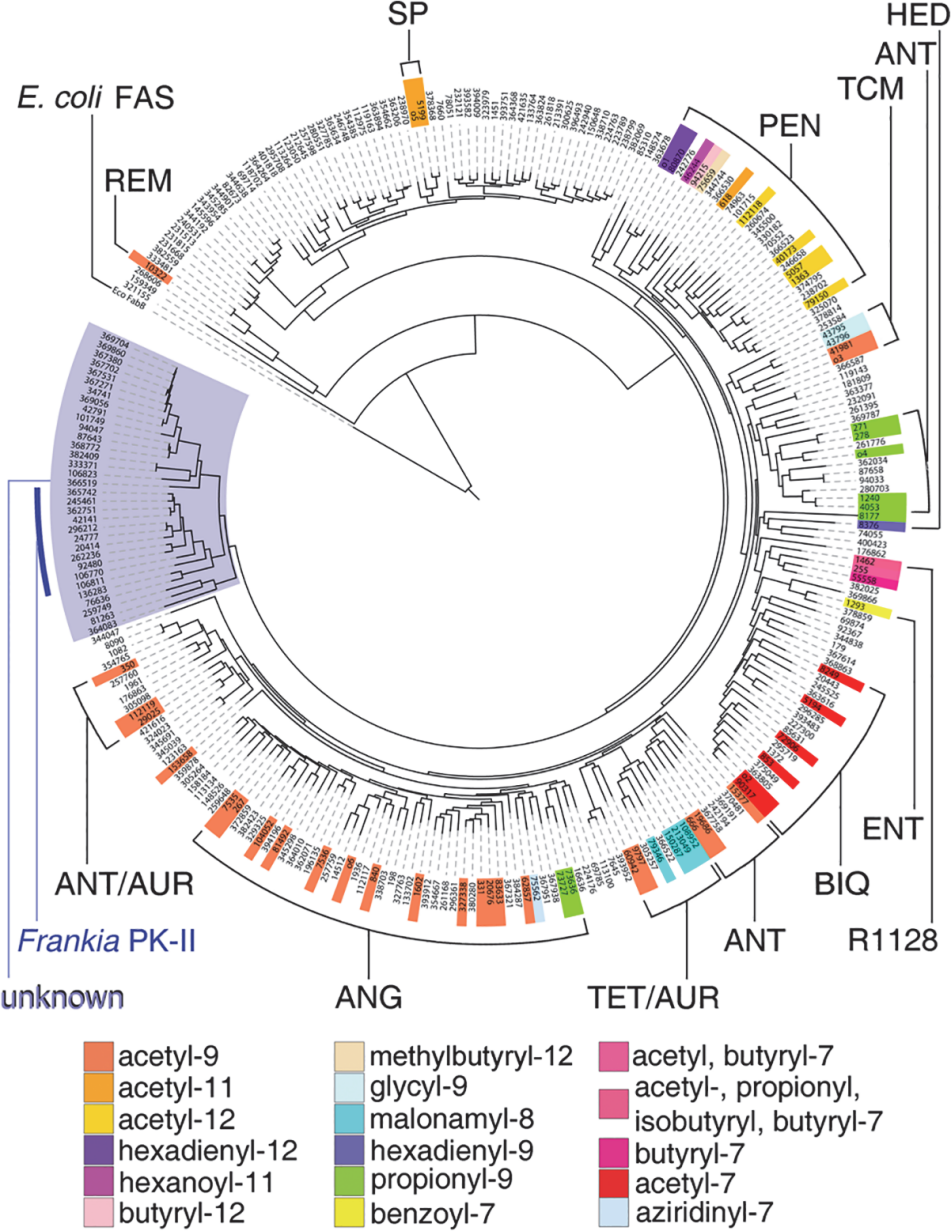
Dendrogram of KSα/β sequences showing the relationship between dendrogramatic position, polyketide subclass, and poly-β-ketone structure. Dendrogram based on multiple alignment of 296 concatenated KSα/β protein sequences illustrating the large uncharacterized clade (left, shaded purple) in which KSα/β pairs from *Frankia* type II polyketide clusters that are the subject of this study (marked with purple bar) are found. KSα/β pairs from previously characterized type II polyketide clusters are colored according to their starter unit and number of extender units (see bottom figure legend, starter/extender colors are listed clockwise as they first appear in the figure). Type II polyketide subclasses are labeled and bracketed. Subclass abbreviations: REM—resistomycin; SP—spore pigment; PEN—pentangular; TCM—tetracenomycin; ANT—anthracycline; HED—hedamycin; R1128—R1128; ENT—enterocin; BIQ—benzoisochromanequinone; TET—tetracycline; AUR—aureolic acid; ANG—angucycline. Other abbreviations: *E*. *coli* FAS—*E*. *coli* fatty acid synthase, which was used as the outgroup.

The ketosynthase α/β dendrogram was generated as follows: The amino acid sequences of all ketosynthase α and ketosynthase β enzymes identified by *Dynamite* were compiled as two separate multi-fasta files using a custom script. Each set was then aligned using Clustal Omega [22] and unconserved N- and C-terminal regions were trimmed based on the multiple sequence alignments to minimize their effects on tree building after constructing the concatenated sequence. Residues corresponding to positions 6–420 of the 424 amino acid actinorhodin KSα, and to positions 1–403 of the 407 amino acid actinorhodin KSβ were retained. Trimmed ketosynthase α/β sequence pairs were concatenated and aligned again using Clustal Omega. A bootstrapped maximum likelihood dendrogram was generated from the alignment using FastTree 2.[23] The dendrogram was visualized and color coded using the Interactive Tree of Life (iTOL) [24] web interface. Ketosynthase I (FabB) from the *Escherichia coli* fatty acid biosynthetic pathway was treated similarly and used to construct a pseudo-dimer sequence that was used as the outgroup. The identities of the 64 studied type II polyketide systems and their starter and extender unit specificities were compiled manually by cross referencing *Dynamite* results with literature, and were color coded by type in iTOL. A high resolution version of the dendrogram in Fig. 3, including bootstrap values, species names, and training set compound names, is available in the S2 Fig.

### Cultivation of bacterial strains

*Frankia alni* ACN14a, *Frankia* sp. EAN1pec, and *Frankia* sp. EuI1c were maintained in Frankia Defined Minimal Medium (FDM) supplemented with the appropriate carbon source(s). *Frankia* sp. CcI3 and *Frankia* sp. EUN1f were maintained in CB Liquid Medium. Both FDM and CB media contain the following: 0.05% w/v NH_4_Cl, 0.02% w/v MgSO_4_•7H_2_O, 0.1% v/v 1000× iron stock solution (0.75% w/v disodium ethylenediaminetetraacetic acid dihydrate, 0.56% w/v FeSO_4_•7H_2_O, and 0.02% w/v Na_2_MoO_4_•2H_2_O). Additionally, FDM medium contains 0.05% w/v Bacto proteose peptone No. 3, 0.01% w/v CaCl_2_•7H_2_O, and 10% v/v 10× phosphate buffer stock solution (0.5 M potassium phosphate buffer, pH 6.5); while CB medium contains 5 g/L sodium pyruvate, 0.16% Bacto proteose peptone No. 3, 0.06% w/v CaCl_2_•7H_2_O, and 10% v/v 10× MOPS-phosphate buffer stock solution (50 mM potassium phosphate, 50 mM MOPS, pH 6.5). The 10× phosphate and phosphate-MOPS buffer stock solutions were added to the media after autoclaving. Fructose (5 g/L) and sodium pyruvate (5 g/L) together were used as the carbon source for *Frankia* sp. EAN1pec and *Frankia alni* ACN14a, and glucose (5 g/L) was used for *Frankia* sp. EuI1c.

### Chromatographic and spectral analysis of *Frankia* extracts

*Frankia* sp. EAN1pec, *Frankia alni* ACN14a, and *Frankia* sp. EuI1c were each cultured in a rotary incubator in 50 mL FDM media, each supplemented separately with five different carbon sources, in 500 mL Erlenmeyer flasks at 28°C, 250 rpm, for two weeks. Carbon sources tested were fructose (5 g/L), sodium pyruvate (5 g/L), fructose (5 g/L) plus sodium pyruvate (5 g/L), sodium succinate (5 g/L), and sodium propionate (5 g/L). The cultures were centrifuged to remove cells. The resulting supernatant was incubated with 5 mL of Amberlite XAD-7 resin, which was washed with 200 mL water. Resin-bound metabolites were eluted with 6 mL of MeOH and the solvent was removed by rotary evaporation. Each sample was re-dissolved in 0.5 mL of 50% aqueous acetonitrile. Ten μL of sample was subjected to LC-MS analysis. Separation was performed by linear gradient elution (0 to 100% solvent B over 12 min) on a C-18 column (Thermo Scientific ODS Hypersil, 5 μm, 150×3 mm). Solvent A: 5% aqueous acetonitrile, 0.1% formic acid; solvent B: 95% aqueous acetonitrile, 0.1% formic acid.

### Isolation of frankiamicin A (4)

The *Frankia* sp. EAN1pec culture was scaled up by stepwise unshaken growth at room temperature in Erlenmeyer flasks with increasing volumes of FDM-fructose/pyruvate media over a period of 6 months. After two to four weeks of growth, cells were collected by centrifugation, homogenized, and transferred to two- to four-fold the original volume of fresh media for the next growth period. After the final growth period, 3.6 L of culture was centrifuged (6000*g*, 15 min) to remove the cells. The resulting supernatant was mixed with 100 mL of Amberlite XAD-7 and the resin was loaded onto a column. The column was washed with water (2 L) and then with 20% aqueous MeOH (1 L). Frankiamicin A and minor congeners were eluted with 50% aqueous MeOH (500 mL). Fractions with red color were collected and concentrated by rotary evaporation. The residue was re-dissolved in 1 mL of water and loaded onto a Sep-Pack C18 column (2 g adsorbent, Varian). The column was washed with 10 mL of water and the desired compounds eluted with 10 mL MeOH. After evaporation of the solvent, the extract was re-dissolved in 2 mL of 10% aqueous MeOH and further purified by HPLC. Purification was performed by linear gradient elution (5 to 95% solvent B over 12 min) on a semi-preparative C-18 column (Thermo Scientific ODS Hypersil, 5 μm, 150×10 mm) at a flow rate of 4 mL/min. Solvent A: water; solvent B: acetonitrile. Frankiamicin A has a retention time of 8 min under these conditions, and was collected manually. Solvent was removed by rotary evaporation and was dried under high vacuum overnight, yielding 3.6 mg of an orange solid.

Supplementation with isotopically-labeled acetate was carried out as follows. Because of its extremely slow growth rate, *Frankia* sp. EAN1pec cells from a previous 0.5 L culture were inoculated into 1 L of fresh FDM-fructose/pyruvate medium and grown in a rotary incubator at 28°C, 250 rpm. An aqueous solution (4 mL) containing 1.0 g of sodium [1,2-^13^C_2_]acetate (99 atom % ^13^C, Aldrich) and 1.0 g of non-labeled sodium acetate was prepared and sterilized by filtration through a syringe filter (pore size: 0.2 μm). Pulse feeding was performed by adding 1 mL of the solution to the culture 2, 5, 8, and 11 days after inoculation. The total concentration of sodium [1,2-^13^C_2_]acetate added was 0.1% w/v. After 17 days, the culture was harvested by centrifugation at 6000*g* for 15 min. The ^13^C-labeled frankiamicin A was isolated from the supernatant as described above. The purified compound was analyzed by ^13^C NMR spectroscopy and the spectrum compared to that of unlabeled compound. The chemical shifts of individual ^13^C signals differed slightly between labeled and unlabeled compounds, likely due to slight conformational differences. To resolve these differences, labeled compound was doped with unlabeled and again analyzed by ^13^C NMR (S8 Fig.).

### Bioactivity assays

Antimicrobial and anticancer assays were conducted by quantifying viability of cells exposed to frankiamicin A (2-fold serial diluted in DMSO) at concentrations ranging from 0–100 μM using an MTT assay [25]. For antimicrobial assays, a liquid culture of each test strain was grown overnight at 37°C in TSB media in a rotary incubator. The resulting culture was diluted 1:100 into fresh media and 100 μL aliquots were transferred to a 96-well plate. Serial diluted compound was added to individual wells and cells were incubated at 37°C for either 6 or 18 h prior to MTT assay. Anticancer assays were conducted using ∼4000 cells incubated overnight at 37°C in 100 μL DMEM media supplemented with 10% FBS, adding serial diluted compound, and incubating for 48 h prior to MTT assay. Assays of *T*. *cruzi* (ATCC 30013) were conducted by growing cells unshaken at 25°C in ATCC Medium 1029 (LIT Medium) for 5 days, diluting 1:10 into fresh media, adding 100 μM frankiamicin A, incubating for an additional 8 days, and assessing cell viability by microscopy using an untreated control for comparison.

## Results and Discussion

### Bioinformatic analysis

We have developed a bioinformatic software package called *Dynamite* that globally identifies and annotates gene clusters responsible for producing three of the most common types of natural products—type I and type II polyketides and non-ribosomal peptides—in all sequences deposited in the NCBI databank to date, rather than in a specific input sequence. Global analysis using *Dynamite* has allowed us to circumscribe all bacterial type II polyketide biosynthetic gene clusters sequenced to date and to systematically compare protein sequences of homologues and distributions of homologous genes across type II polyketide gene clusters in search of proteins and gene clusters with atypical features.

After identifying all 296 putative bacterial type II polyketide gene clusters present in the NCBI databank as of December 2013, we carried out further comparative analyses of genes within these clusters to identify those with unique sequence characteristics. We began by conducting dendrogramatic analysis of the sequences of KSα/β, the heterodimeric enzyme responsible for biosynthesis and chain length control of the poly-β-ketone precursors of all bacterial type II polyketides. Reasoning, as previous studies [26] had, that KSα/β sequences would co-vary with the poly-β-ketone chain lengths/structures they produce, we generated a dendrogram of concatenated KSα/β amino acid sequences from all 296 type II polyketide clusters identified by *Dynamite* (Fig. 3, S1 Table), including the 64 training set gene clusters responsible for biosynthesis of natural products with known poly-β-ketone lengths, structures, and cyclized core structures (Fig. 3, S2 Fig., colored by starter unit/extender unit number). This analysis revealed strong correlations between the positions of training set KSα/β sequences in the dendrogram and both poly-β-ketone chain length/structure and type II polyketide structural subclass. While most branches of the dendrogram harbor at least one training set KSα/β sequence, we identified a large, diverged clade comprised entirely of KSα/β sequences from uncharacterized type II polyketide gene clusters (Fig. 3, left, highlighted in purple). Within this clade were a closely related set of 11 KSα/β sequences from the genomes of 10 *Frankia* species (Fig. 3, marked with purple bar), a group of nitrogen-fixing Actinobacterial plant root endophytes [27]. According to our bioinformatic analysis and that of others [28], *Frankia* genomes [29] harbor a large and diverse set of polyketide and non-ribosomal peptide natural product gene clusters (see S2 and S3 Tables, respectively, for a list of all natural product gene clusters identified using *Dynamite* in the *Frankia* genomes analyzed, and for further information on these genomes). However, only three *Frankia* natural products—the related pentangular polyketides G-2A and G-2N [30] and the calcium-binding antibiotic, demethyl cezomycin (frankiamide) [31]—have been structurally characterized to date; and there have been no reported examples thus far in which a functional link between a *Frankia* natural product and the gene cluster responsible for its production has been established. Thus, *Frankia* are understudied in regard to their genetic capacity to produce natural products.

**Fig 4:**
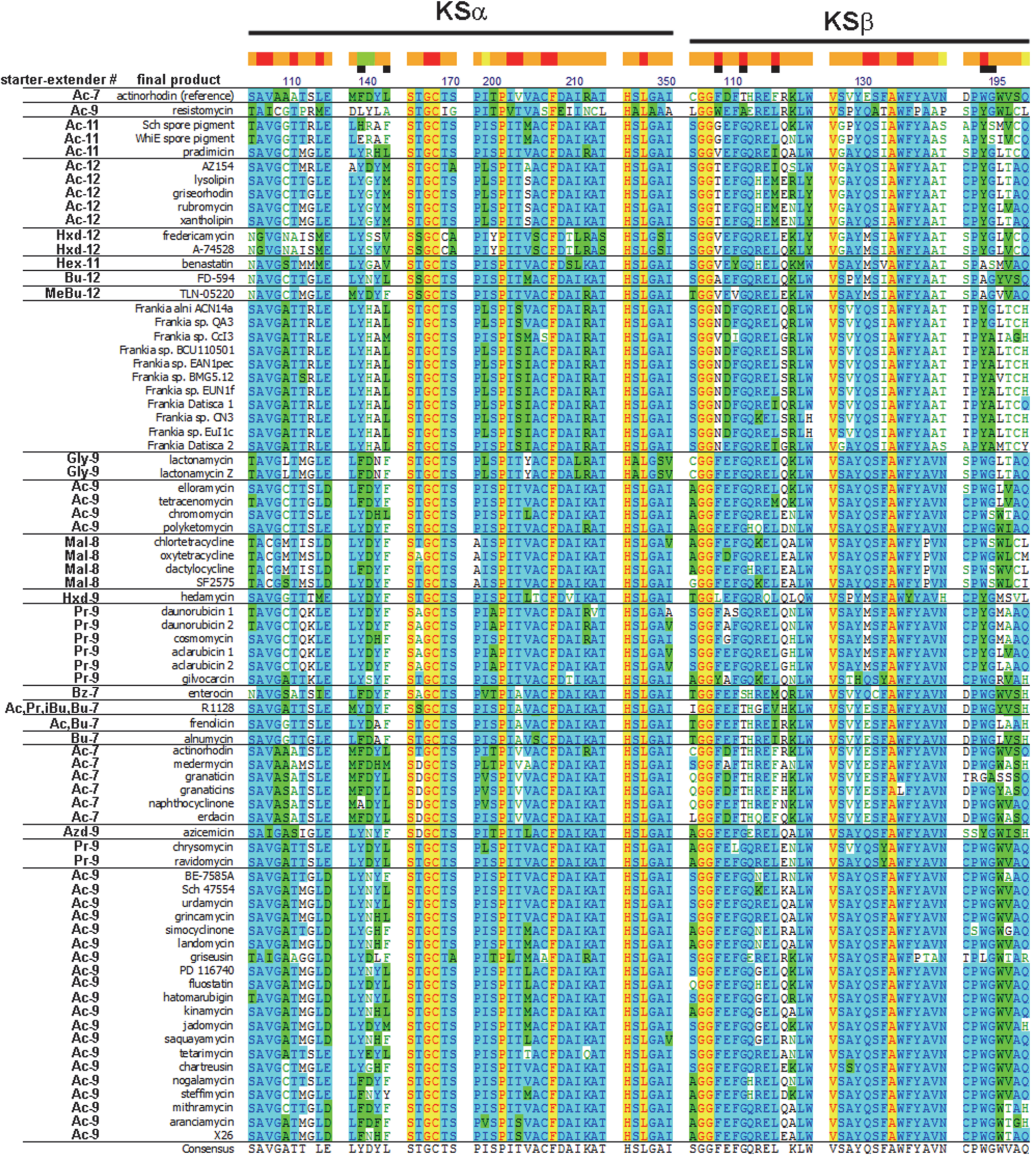
Multiple sequence alignment of training set and *Frankia* KSα/β active site residues. Eight regions of KSα/β protein sequence from the 64 KSα/β training set members and eleven *Frankia* KSα/β sequences that are predicted to be in the closest proximity to the active site based on the X-ray crystal structure of the actinorhodin (*act*) KSα/β are shown. The five regions that lie within KSα and the three that lie within KSβ are noted by labeled black bars at the top of the figure. Predicted proximity to the active site is shown as a heat map at the top of the figure (red residues line the active site pocket, orange residues are within 4Å of the residues that line the active site, yellow residues are within 6Å, and green residue are within 8Å. Black squares immediately below the heat map mark the seven residues previously proposed to be responsible for product specificity. Residues are numbered using *act* numbering. Training set product names and *Frankia* cluster names are given to the left. Starter unit and number of extender units of training set systems appear on the far left. Ac—acetyl, Pr—propionyl, Mal—malonamyl, Gly—glycyl, Bu—butyryl, iBu—isobutyryl, Azd—aziridinyl, Hxd—hexadienyl, Hex—hexanoyl, MeBu—2-methylbutyryl, Bz—benzoyl.

Studies of the actinorhodin KSα/β [10] crystal structure identified the residues comprising the enzyme active site and proposed that seven of these (F140, L143 of KSα and F109, T112, F116, W194, and G195 of KSβ) might be responsible for determining poly-β-ketone chain length, including three residues (F109, T112, F116 of KSβ) that had previously been shown through mutagenesis to be directly involved in chain length determination [11]. In an attempt to gain further insight into the poly-β-ketone chain length produced by the *Frankia* KSα/β enzymes, we compared the identities of the proposed chain length determining residues and other residues in proximity to the active site of *Frankia* KSα/β with those of all training set KSα/β via multiple sequence alignment. Overall, predicted *Frankia* KSα/β active site residues were most similar to those of training set members producing poly-β-ketone intermediates of at least 24 carbons, particularly at positions 133, 139, and 140 of KSβ, where all training set sequences responsible for making products of at least 24 carbons had I/V, A, S/T, respectively (Fig. 4). However there were some notable exceptions, such as the unique and well-conserved A110 and S204 of KSα and N109, D110, R118, V129, T192, A195 of KSβ.

Because of the distinct sequence characteristics of members of this clade and the lack of KSα/β sequences from the training set within the clade, it was not possible to predict with certainty from KSα/β sequence analysis which poly-β-ketone chain length/structure was produced by these enzymes, or the structural subclass to which their cyclized products belong.

**Fig 5:**
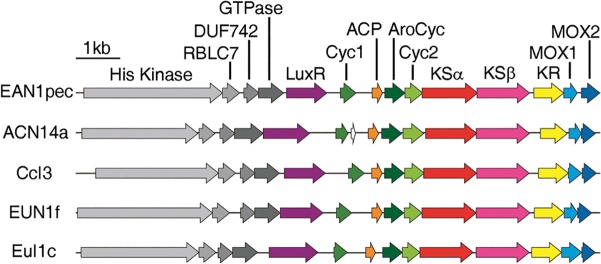
Gene synteny in representative *Frankia* type II polyketide gene clusters. Homologous genes appear in the same color. Species abbreviations: EAN1pec—*Frankia* sp. EAN1pec; ACN14a - *Frankia* alni ACN14a; CcI3—*Frankia* sp. CcI3; EUN1f - *Frankia* sp. EUN1f; EuI1c - *Frankia* sp. EuI1c. Gene function abbreviations: His Kinase—histidine kinase; RBLC7—road block LC7 family protein; DUF742—domain of unknown function 742; GTPase—Ras family GTPase; LuxR—LuxR family transcriptional regulator; Cyc1—TcmI-like polyketide cyclase, AroCyc—TcmN-like aromatase/cyclase, Cyc2—TcmJ-like polyketide cyclase, KR—ketoreductase; MOX1—PdmH-like putative monooxygenase, MOX2—PdmI-like putative monooxygenase.

*Dynamite* analysis of the proteins encoded by genes adjacent to the *Frankia* KSα/β genes revealed seven other proteins characteristic of bacterial type II polyketide biosynthesis: an acyl carrier protein (ACP), three cyclases, two putative monooxygenases, and a ketoreductase; as well as five proteins with homology to those involved in signal transduction and regulation of gene expression. We observed nearly complete synteny and a high degree of sequence similarity (Fig. 5) among homologous genes from each *Frankia* species, suggesting that the clusters make the same or highly similar products. All 14 genes in each cluster are also co-directional, suggesting that they comprise a single operon. No additional conserved proteins with homology to known natural product biosynthetic or regulatory proteins were found encoded in the regions flanking these *Frankia* type II polyketide gene clusters.

Sequence comparison of each putative biosynthetic protein in the *Frankia* clusters to proteins from type II polyketide training set clusters revealed a high degree of similarity between each putative *Frankia* biosynthetic protein and proteins from pentangular and tetracenomycin subclass products (summarized in S4 Table), suggesting that the *Frankia* clusters either produce a compound from one of these subclasses or from a novel, but biosynthetically closely related subclass. The conserved set of three cyclases characteristic of pentangular and tetracenomycin subclass products—a monodomain aromatase/cyclase homologous to the N-terminal domain of TcmN [32,33], a cyclase with predicted cupin-like fold homologous to TcmJ [34], and a cyclase with predicted ferredoxin-like fold homologous to TcmI [35]—were present in the clusters. Support for the tentative placement of the *Frankia* clusters within the pentangular subclass came from sequence analysis of the two putative monooxygenases and the ketoreductase found in each cluster. Homologues of each of the two putative monooxygenases are found encoded adjacent to each other in each pentangular training set cluster, whereas only a single more distantly related homologue is present in tetracenomycin subclass clusters; and the *Frankia* ketoreductases are highly similar to tailoring ketoreductases known to reduce the C-6 position of the polyketide in pentangular pathways [36,37], but which are absent from tetracenomycin subclass clusters.

Biosynthesis of the polyketide core structures of seven of the sixteen pentangular, tetracenomycin, or related unique training set compounds are known or predicted to be initiated by incorporation of a non-acetate starter unit [38,39–42]. In each case, a type III ketosynthase or stand-alone adenylation domain is present in the gene cluster. The absence of homologues of either of these genes in the *Frankia* cluster suggests that each produces an acetate-primed polyketide product.

In contrast to most training set type II polyketide clusters, which encode a number of additional tailoring enzymes, the *Frankia* clusters lack additional putative tailoring enzymes other than the ketoreductase, suggesting that their product represents a minimally modified aromatic polyketide.

Taken together, bioinformatic analysis suggests that the *Frankia* clusters in question biosynthesize a product made from an acetate primed poly-β-ketone of at least 24 carbons, are biosynthetically and structurally related to pentangular and tetracenomycin subclass compounds and are more similar to pentangular subclass compounds. However, the KSα/β sequences from these clusters have diverged sufficiently from those of training set members to preclude accurate chain length prediction. In order to establish a sequence-function relationship between this group of orphan gene clusters and their product, we sought to isolate and structurally characterize compounds made by this group of gene clusters.

### Chromatographic and spectral analysis of *Frankia* extracts

**Fig 6:**
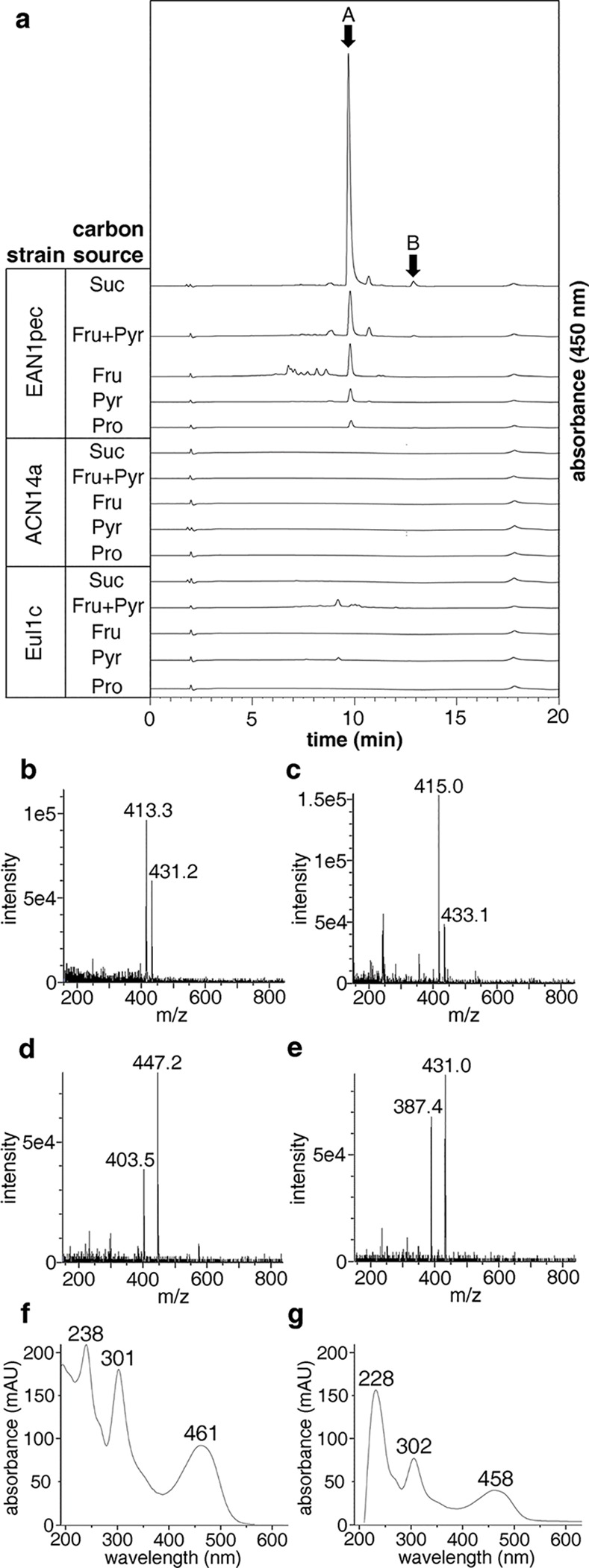
UV-visible and mass spectral analysis of *Frankia* extracts and metabolites. (a) HPLC analysis of extracts from the three *Frankia* species grown using different carbon sources, and showing the presence of the major compound (labeled A) and the minor compound (labeled B). (b-g) ESI-MS analysis in positive and negative ionization modes and photodiode array (PDA) spectra of the major and minor peaks (data collected from 9.4–9.7 min, 12.7–12.9 min, respectively). (b) major peak, positive mode (M + H − H_2_O and M + H − 2 H_2_O); (c) minor peak, positive mode (M + H, M + H − H_2_O); (d) major peak, negative mode (M − H, M − H − CO_2_); (e) minor peak, negative mode (M − H, M − H − CO_2_); (f) PDA spectrum of the major peak; (g) PDA spectrum of the minor peak.

Five *Frankia* strains (*Frankia alni* ACN14a, *Frankia* sp. CcI3, *Frankia* sp. EAN1pec, *Frankia* sp. EuI1c, and *Frankia* sp. EUN1f), each harboring a single copy of the gene cluster in question, were selected for characterization. Each was first grown in small scale in the recommended media (see Experimental Section). While the growth rates of all *Frankia* species examined were quite low (doubling of wet cell weight occurred every 2 to 3 weeks), those of *Frankia* sp. CcI3 and *Frankia* sp. EUN1f were the lowest. These two strains were therefore not pursued further. Because media composition is known to have a profound impact on natural product production [43], each of the three remaining strains (*Frankia alni* ACN14a, *Frankia* sp. EAN1pec, and *Frankia* sp. EuI1c) was cultured in small scale (50 mL) in five different media that differed with respect to the carbon source(s) (fructose, pyruvate, fructose + pyruvate, succinate, and propionate were employed). Extracts from each of these fifteen strain/media combinations were obtained by adsorption onto and elution from Amberlite XAD-7 resin, and were analyzed by HPLC-PDA/MS. While extracts from *Frankia alni* ACN14a and *Frankia* sp. EuI1c showed no major uv-visible or mass spectral peaks in any of the five media, the extracts obtained from *Frankia sp*. EAN1pec showed one major peak [r.t. = 9.7 min, ESI-positive m/z = 413.3 (M + H- 2H_2_O), 431.2 (M + H—H_2_O); ESI-negative m/z = 403.5 (M—H—CO_2_), 447.2 (M—H)] and one minor peak [r.t. = 12.9 min, ESI-positive m/z = 415.0 (M + H—H_2_O), 433.1 (M + H); ESI-negative m/z = 387.4 (M—H—CO_2_), 431.0 (M—H)], each with absorption in the visible range (Fig. 6A-E). The uv-visible spectra of the major and minor compounds closely resembled each other, displaying peaks at ∼300 and ∼460 nm (Fig. 6F, G), suggesting that they are congeners. Production of these two compounds was highest with succinate as the sole carbon source, reached significant levels with either fructose alone or with fructose and pyruvate, and was low with either pyruvate or propionate alone (Fig. 6A). Extracts containing large amounts of these compounds displayed a deep red color not present in *Frankia alni* ACN14a or *Frankia* sp. EuI1c extracts. The lack of detectable products in *Frankia alni* ACN14a and *Frankia* sp. EuI1c is likely due to their natural product biosynthetic gene clusters being cryptic—transcriptionally inactive—under the culture conditions used [44,45]. The high resolution ESI-TOF MS of the major compound (*m/z*: [M—H] calculated for C_24_H_15_O_9_ 447.0716; found 447.0709), which we named frankiamicin A, supported the notion that the KSα/β from the clusters in question produces a 24 carbon aromatic polyketide. The minor compound, whose parent mass is 432, was named frankiamicin B. After obtaining these results, we next turned our attention to isolation and structure elucidation of frankiamicin A in order to provide further experimental support for the product specificities of the KSα/β and the cyclases in the *Frankia* clusters.

### Isolation and structure elucidation of frankiamicin A

Cultures of *Frankia* sp. EAN1pec were scaled up in a stepwise fashion to 3.6 L total volume from an initial seed culture over a 6 month period, and 3.6 mg of frankiamicin A was isolated from the resulting culture broth by a three step chromatographic procedure (see Experimental Section for details). Frankiamicin A is an orange amorphous solid that is soluble in water and DMSO. ^1^H and ^13^C NMR spectral data (Table 1 and S3, S4 Figs.) reveal the presence of 10 proton and 24 carbon signals, consistent with high resolution MS analysis. Nineteen of the 24 carbon signals present in the ^13^C NMR spectrum have chemical shifts between δ 100 and 170 ppm, consistent with aromatic carbon atoms; and two carbonyl resonances were observed at 189.5 and 181.8 ppm, consistent with frankiamicin A being an aromatic polyketide compound with a quinone moiety. The ^1^H NMR spectrum of frankiamicin A displays four aromatic proton signals, one aliphatic proton signal with an adjacent hydroxyl group, one pair of geminal protons (2.98 and 2.81 ppm, *J* = 15.6 Hz), one aromatic methyl group (2.58 ppm), and two exchangeable protons (11.37 and 5.26 ppm). ^1^H-^1^H COSY (S5 Fig.) NMR coupling constants demonstrate connectivity between H-5 (4.52 ppm) and both protons at C-6 (2.98, 2.81 ppm) and between H-5 and the exchangeable proton at 5.26 ppm. Two aromatic protons (H-10, H-12; 6.60 and 7.17 ppm, respectively) are coupled to each other with coupling constant of 2.1 Hz, suggesting a *meta* relationship. The ^1^H NMR signals of the remaining two aromatic protons and the methyl group were singlets.

**Table 1:** NMR spectroscopic data (DMSO-*d*_*6*_) for frankiamicin A (4).

**position**	**δ**_H_ **(multiplicity, *J* in Hz)**	**δ**_C_	***J*** ^13^ **C-** ^13^ **C** ^[^ ^a^ ^]^
1		164.3^[^ ^b^ ^]^	^[^ ^b^ ^]^
2		118.0	62.3
3		143.5	45.6
4	6.58 (s)	116.3	59.8
4a		143.0	60.6
5	4.53 (dt, 8.9, 4.6)	66.3	37.6
6	2.83 (dd, 15.8, 8.9)	29.5	37.7
	2.98 (dd, 15.8, 4.6)		
6a		128.4	69.3
7		158.5	69.1
7a		112.0	56.9
8		189.4	56.0
8a		109.2	64.9
9		165.4^[^ ^b^ ^]^	^[^ ^b^ ^]^
10	6.60 (d, 2.1)	107.7	66.8
11		166.1^[^ ^b^ ^]^	^[^ ^b^ ^]^
12	7.18 (d, 2.1)	108.6	64.1
12a		135.5	64.5
13		181.8	55.1
13a		130.1	55.5
14	9.20 (s)	120.0	56.2
14a		142.6	56.5
14b		115.7	63.9
15	2.58 (s)	23.8	43.0
16		171.4	64.4
5-OH	5.27 (d, 4.6)		

[a] Coupling constants in Hz, observed by [1,2-^13^C_2_]acetate feeding.

[b] Obscured by overlapping.

**Fig 7:**
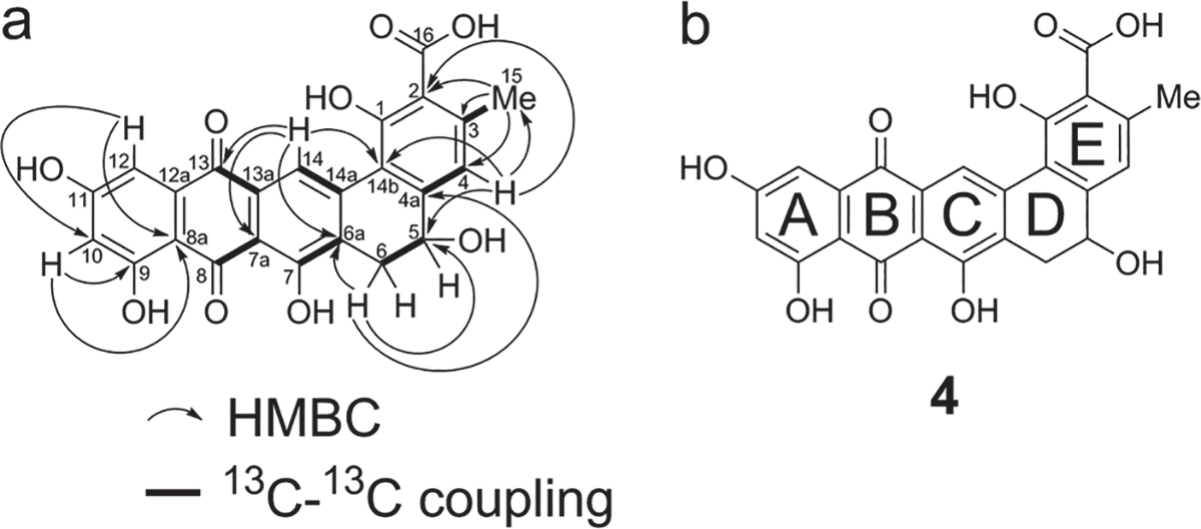
Structural analysis and elucidation of frankiamicin A (4). a) HMBC correlations and ^13^C-^13^C couplings observed through [1,2-^13^C_2_]acetate feeding. b) structure of frankiamicin A.

Single and multiple bond C-H correlations were elucidated by HMQC and HMBC experiments, respectively. The HMQC spectrum (S6 Fig.) was used to assign the signals of the seven carbon atoms that are directly connected to protons. ^13^C chemical shifts indicate that three of these (C-15, C-5, and C-6) are *sp* ^*3*^ hybridized, and four (C-4, C-10, C-12, and C-14) are *sp* ^*2*^ hybridized. The HMBC spectrum (Fig. 7A, S7 Fig.) showed that one of the carbonyl carbons (C-13, 181.8 ppm) has long range connectivity to two aromatic protons (H-12 and H-14). HMBC correlations from H-14 and H-6 to C-6a, and from H-14 and H-4 to C-14b were also observed, suggesting the structure of rings A-D of frankiamicin A. Further HMBC correlations from H-15 to C-2, C-3, and C-4; and from H-4 to C-2 and C-15 placed the methyl group at C-3, and allowed us to propose the structure of frankiamicin A as **4** (Fig. 7B).

Since C-H correlations for eight carbon atoms (C-1, C-7, C-8, C-11, C-12a, C-13a, C-14a, and C-16) could not be observed through either HMQC or HMBC analyses, a ^13^C enrichment study using [1,2-^13^C_2_]acetate was carried out to obtain additional information on carbon atom connectivity. We grew *Frankia* sp. EAN1pec cells obtained from a 0.5 L initial culture in 1 L of fresh media for 17 days while supplementing with 250 mg of sodium [1,2-^13^C_2_]acetate on days 2, 5, 8, and 11 to obtain frankiamicin A that was partially labeled with intact [1,2-^13^C_2_]acetate units. The resulting compound (1.3 mg) was purified and analyzed by ^13^C NMR spectroscopy (S8 Fig.). In the spectrum obtained, all carbon signals are doublets that correspond to singlet signals in the ^13^C spectrum of the unlabeled compound. The ^13^C-^13^C spin couplings observed originate from intact incorporation of [1,2-^13^C_2_]acetate units into frankiamicin A, while ^13^C-^13^C spin couplings between two different acetate units are not observed due to the low incorporation ratio of labeled acetate. Analysis of coupling constants (Fig. 7A, Table 1, right column) clearly elucidated connectivity of C3 and C15, C4 and C4a, C5 and C6, C6a and C7, C7a and C8, C13 and C13a, and C14 and C14a. The four signals corresponding to C-14b, C-1, C-2, and C-16 are all doublets with similar coupling constants, indicating that these four carbon atoms are collectively derived from incorporation of two intact acetate units. Similarly, the remaining six carbon atoms, C-8a, C-9, C-10, C-11, C-12, and C-12a, whose coupling constants are also similar, are collectively derived from incorporation of three intact acetate units. The results of 1-D and 2-D NMR studies of the unlabeled compound together with analysis of the ^13^C spectrum of the labeled compound provide strong support for the proposed structure of frankiamicin A as the 24-carbon pentangular polyketide **4**.

**Fig 8:**
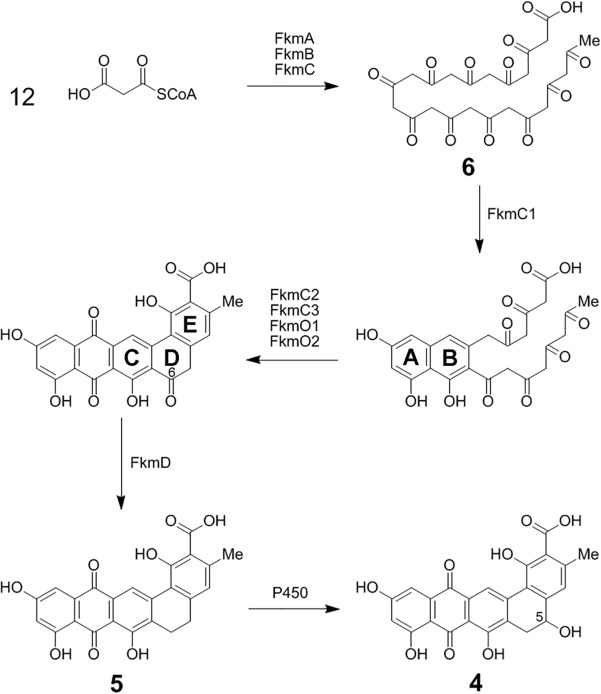
Proposed frankiamicin A biosynthetic pathway. The minimal polyketide synthase FkmABC catalyze conversion of 12 malonyl-CoA units to the 24 carbon poly-β-ketone **6**; TcmN-like aromatase/cyclase FkmC1 catalyzes closure and aromatization of rings A and B; FkmC2, C3, O1, and O2 catalyze closure of the C, D, and E rings, aromatization of the C and E rings, and oxygenation of the B ring; FkmD catalyzes reduction of the C-6 ketone to form G-2A (**5**); and a P450 monooxygenase catalyzes C-5 hydroxylation to generate frankiamicin A (**4**).

The structure of **4** together with the fact that *Frankia* sp. EAN1pec harbors only a single type II polyketide cluster strongly support the idea that **4** is produced by this cluster. Consistent with this notion, a recent proteomic study of *Frankia* sp. EAN1pec grown in similar culture conditions to those we used, detected peptide fragments corresponding to proteins encoded by this gene cluster [28]. The highly conserved gene composition and arrangement, and the high degree of sequence similarity observed among the group of *Frankia* type II polyketide gene clusters analyzed suggests that each of them is responsible for production of **4** or a closely related, minimally tailored 24-carbon pentangular polyketide. Thus, the *Frankia* KSα/β enzymes represent a new group of 24 carbon poly-β-ketone synthesizing KSα/β that has diverged in sequence from homologues that produce the same intermediate. Furthermore, the structure of **4** strongly supports the idea that the immediate tailoring enzymes in the *Frankia* clusters collectively function to produce a pentangular, rather than a tetracenomycin, or atypical polyketide core structure. Interestingly, an engineered compound JX134 [45], which is identical in structure to **4**, was produced by heterologous expression of a set of 9 pradimicin biosynthetic genes, including 8 that are homologues of genes in the *Frankia* clusters, supporting the idea that homologous genes in the two clusters are functionally equivalent. The minor congener observed during initial LC-MS analysis, which we named frankiamicin B, was present in sufficiently small quantities (1% of frankiamicin A) to preclude NMR structural analysis, but is likely G-2A (**5**, Fig. 8), the 5-deoxy derivative of frankiamicin A that was previously isolated, together with its C-2 decarboxylated congener G-2N, from *Frankia* sp. G2 [31]. This, together with our comparative genomic analysis of the *Frankia* clusters, suggests that the ability to produce G-2A and congeners is well-conserved among *Frankia* species, and that G-2A and G-2N are produced in *Frankia* sp. G2 by a gene cluster analogous to those we identified in sequenced *Frankia* genomes.

### Proposed biosynthesis of frankiamicin A

To aid in discussion of the proposed functions of genes in the *Frankia* sp. EAN1pec cluster, we have assigned each gene a systematic name. These names, their corresponding locus tags, GI numbers, and proposed functions are summarized in Table 2. An expanded table (S4 Table) containing comparative genomic information on all homologous gene clusters from five *Frankia* species and on all pentangular and tetracenomycin training set clusters can be found in the Supporting Information.

**Table 2:** Frankiamicin (*fkm*) cluster genes, homologues, and proposed functions.

			**Homologues**				
**Gene**	**Locus Tag**	**GI#**	***tcm***	***pdm***	***ben***	**Annotation**	**Proposed Function**
*fkmR1*	FranEAN1_2384	158314214	*---*	*---*	*---*	signal transduction histidine kinase	Cluster regulation by signal transduction
*fkmR2*	FranEAN1_2385	158314215	*---*	*---*	*---*	Roadblock/LC7 family protein	Cluster regulation by signal transduction
*fkmR3*	FranEAN1_2386	125314216	*---*	*---*	*---*	protein of unknown function DUF742	Cluster regulation by signal transduction
*fkmR4*	FranEAN1_2387	125314217	*---*	*---*	*---*	GTPase	Cluster regulation by signal transduction
*fkmR5*	FranEAN1_2388	125314218	*---*	*---*	*---*	LuxR family transcriptional regulator	cluster regulation
*fkmC3*	FranEAN1_2389	125314219	*tcmI*	*pdmK*	*benE*	polyketide synthesis cyclase	D, E ring cyclization
*fkmC*	FranEAN1_2390	125314220	*tcmM*	*pdmC*	*benC*	acyl carrier protein	acyl carrier protein
*fkmC1*	FranEAN1_2391	125314221	*tcmN*	*pdmD*	*benH*	cyclase/dehydrase	A, B ring cyclization
*fkmC2*	FranEAN1_2392	125314222	*tcmJ*	*pdmL*	*benD*	cupin fold family cyclase	C ring cyclization
*fkmA*	FranEAN1_2393	125314223	*tcmK*	*pdmA*	*benA*	β-ketoacyl synthase	ketosynthase α
*fkmB*	FranEAN1_2394	125314224	*tcmL*	*pdmB*	*benB*	β-ketoacyl synthase	ketosynthase β
*fkmD*	FranEAN1_2395	125314225	*---*	*pdmG*	*benL*	ketoreductase	C-6 reduction
*fkmO1*	FranEAN1_2396	125314226	*---*	*pdmH*	*benH*	putative ABM monooxygenase	quinone formation/D, E ring cyclization
*fkmO2*	FranEAN1_2397	125314227	*---*	*pdmI*	*benJ*	Putative ABM monooxygenase	quinone formation/D, E ring cyclization

In light of the structure of **4** and the gene composition of the *Frankia* type II polyketide clusters analyzed here, the biosynthesis of the frankiamicin polyketide core structure appears to follow closely that proposed for pradimicin [37], which shares the same core structure. The FkmA, FkmB, and FkmC proteins correspond to the KSα, KSβ, and ACP minimal polyketide synthase genes, respectively. We propose that these 3 proteins act in concert to produce the 24 carbon poly-β-ketone intermediate **6** via 11 cycles of Claisen condensation (Fig. 8).

The three cyclases found in the cluster, FkmC1, FkmC2, and FkmC3, are homologous to TcmN/PdmD, TcmJ/PdmL, and TcmI/PdmK, respectively, from tetracenomycin and pradimicin pathways. Homologues of these three cyclases are invariably present in type II polyketide gene clusters belonging to the pentangular and tetracenomycin subclasses. Precise assignment of the substrates and products of cyclases and other immediate tailoring enzymes is notoriously difficult due both to the high reactivity of the poly-β-ketone-containing cyclization intermediates; and to the likelihood that these enzymes form complexes with the minimal polyketide synthase in which they act interdependently, and serve both catalytic and structural roles [12,46–48]. Cyclase functions are usually inferred from *in vitro* and *in vivo* analysis of shunt metabolites accumulated when the minimal polyketide synthase and specific sets of cyclases are present. Through such studies, homologues of FkmC1, TcmN [32] and PdmD [37], have been shown to cyclize and aromatize both the A and B rings of the nascent aromatic polyketide. Predicted cupin-like fold cyclases TcmJ [34] and PdmL [37], homologues of FkmC2; and predicted ferredoxin-like fold cyclases TcmI [35] and PdmK [37], homologues of FkmC3, were each shown to be essential for efficient production of the fully cyclized aromatic polyketide cores in their respective pathways. TcmI was shown *in vitro* to catalyze closure of the tetracenomycin D ring [35]. Taken together, comparative analysis and literature precedent suggest that FkmC2 and its homologues are involved in efficient closure and aromatization of the C ring; and FkmC3 and its homologues are involved in efficient closure of the D ring, and possibly in cyclization and aromatization of the E ring in pentangular pathways (Fig. 8, Table 2).

FkmO1 and FkmO2, two antibiotic biosynthesis monooxygenase (ABM) superfamily members, are also present in the cluster. Homologues of both are present in, and encoded by adjacent co-directional genes in all training set pentangular clusters. The closest characterized homologues of FkmO1 and FkmO2 are PdmH and PdmI, respectively, from the pradimicin pathway. Heterologous expression studies demonstrated that PdmH is required for formation of rings C through E of the pentangular core structure whereas PdmI was shown to be non-essential [37]. More distantly related ABM superfamily members from type II polyketide pathways whose reactions have been characterized *in vitro*, such as TcmH [49], ActVA-ORF6 [50,51], AknX [52], and SnoaB [53] catalyze oxygenation of the anthrone B ring to generate a quinone. This led to the suggestion that PdmH catalyzes an analogous reaction in pradimicin biosynthesis [37]. However, all B ring oxygenation reactions characterized *in vitro* thus far occur as tailoring steps after the aromatic core structure is formed, whereas PdmH is proposed to act in concert with cyclases PdmL and PdmK at some point amid cyclization of rings C through E. Interestingly, it was noted during structural studies of cyclase TcmI [54] that this protein and anthrone oxygenase ActVA-ORF6 [51] have strong topological similarity and share the ferredoxin-like fold. This suggests an evolutionary, and possibly a functional link, between TcmI-like cyclases and ABM superfamily members. It is therefore possible that ABM superfamily members FkmO1 and FkmO2 and their homologues may be involved in pentangular polyketide cyclization. In light of the conservation of homologues of both proteins in the eleven pentangular clusters sequenced thus far [5,6,39–42,55–60] but not in tetracenomycin class clusters; and the conserved adjacent co-directional arrangement of their encoding genes, we hypothesize that both FkmO1 and FkmO2 and their homologues are immediate tailoring enzymes that may be involved in B ring oxygenation and/or E ring cyclization and aromatization (Fig. 8, Table 2). Further studies are needed to test this hypothesis and to determine the order in which cyclases FkmC2 and FkmC3, and ABM family proteins FkmO1 and FkmO2 act; and to delineate the catalytic versus structural roles these enzymes may serve in pentangular core structure formation.

The gene product of FkmD is homologous to ketoreductases from pentangular pathways such as BenL [36] and PdmG [37] from benastatin and pradimicin pathways, respectively. Homologues of FkmD are invariably present in pentangular clusters. Gene disruption and heterologous expression studies indicate that BenL and PdmG both catalyze reduction of the ketone at C-6 of the pentangular core structure. This occurs as a tailoring step after polyketide cyclization and B ring quinone formation. In studies of pradimicin biosynthesis, expression of PdmG along with the minimal polyketide synthase, cyclases, and monooxygenase led to a fully reduced C5-C6 bond, demonstrating that C-6 dehydration and a second reduction at C-6 occur. Most pentangular polyketides whose biosynthesis has been studied thus far have a fully reduced C5-C6 bond. Whether the second reduction is also catalyzed by FkmD and its homologues awaits further study. However, we hypothesize based on the lack of another conserved reductase within pentangular clusters that FkmD catalyzes C-6 ketoreduction, C5 dehydration and aromatization, and C-6 enoylreduction to generate G-2A (**5**) (Fig. 8). The observation that LanV, a ketoreductase from the landomycin pathway, a type II polyketide of the angucycline subclass and homologue of FkmD, catalyzes both C-6 ketoreduction and C5 dehydration/aromatization of the angucycline core structure [61] lends support to this hypothesis.

The final step in the proposed biosynthesis of frankiamicin A (**4**) is C-5 hydroxylation. A cytochrome P450 monooxygenase PdmJ was shown to introduce a hydroxyl group at the C-5 position in the biosynthesis of pradimicin [62]. This modification is not conserved in pentangular pathways, but also likely occurs in FD-594 biosynthesis [42] based on the presence of a C-5 hydroxyl in the structure and a close homologue of PdmJ in the cluster. Surprisingly, a likely candidate for C-5 hydroxylation of G-2A to generate frankiamicin A is absent from both the *Frankia* sp. EAN1pec cluster and its homologues in other *Frankia* genomes. While it is unclear from bioinformatic analysis which enzyme might be responsible for C-5 hydroxylation in *Frankia* sp. EAN1pec, or whether this modification is conserved in the *Frankia* type II polyketide pathways analyzed, several P450 enzyme candidates, including nearby Franean1_2408, are encoded in the *Frankia* sp. EAN1pec genome.

### Signal transduction and regulatory proteins in the *fkm* cluster

The frankiamicin gene cluster encodes several proteins (FkmR1-FkmR5) with homology to proteins involved in transcriptional regulation and signal transduction. Among these, FkmR5 is homologous to members of the LuxR family of transcriptional regulators [63], which are commonly found at the edges of natural product biosynthetic gene clusters and have been found to function as cluster-specific regulators (CSRs) that can either activate [64,65] or repress [66,67] transcription of natural product gene clusters. The four gene cassette *fkmR1-fkmR4* is homologous to a conserved set of genes termed the conservon that are present in a number of Actinobacterial genomes. The existence of the conservon was first noted after sequencing the *Streptomyces coelicolor* A3(2) genome, which harbors 13 copies of this gene cassette [68]. Subsequent genetic and biochemical studies of one *S*. *coelicolor* conservon, *cvn9*, showed that these proteins form a membrane associated complex comprised of an integral membrane histidine kinase, Ras-like GTPase, and two accessory proteins. The Cvn9 complex was shown to be involved in regulation of morphological differentiation and antibiotic production. It was suggested that conservon homologues act as signal transducers that receive environmental signals and stimulate intracellular responses [69]. The presence of the *fkmR1-R4* conservon within the *fkm* operon suggests that it transduces an extracellular signal into an intracellular response that leads to activation or repression of frankiamicin cluster expression, possibly via interaction with FkmR5. To our knowledge, homologues of *fkmR1-R4* do not occur as part of any natural product biosynthetic gene clusters studied to date, suggesting that the *fkm* cluster may be regulated differently than other natural product clusters.

### Bioactivity assays of frankiamicin A

Unlike the vast majority of other type II polyketide natural products studied to date, which were identified through bioactivity-guided approaches, frankiamicin A was discovered through a bioinformatics-guided approach. Therefore, nothing was known *a priori* about its bioactivity. Compared to frankiamicin A, many other members of the pentangular type II polyketide subclass with diverse bioactivities such as pradimicin [5,6,37], fredericamycins [39], lysolipin [58], and A-74528 [41] undergo extensive tailoring modifications that substantially alter the polyketide core structure. Several bioactive compounds that have less substantial structural modifications to the polyketide core, and are therefore more similar to frankiamicin A, are known. These include the antibacterial BE-39589 group [70], the phosphodiesterase inhibitor KS-619-1 [71], and the glutathione *S*-transferase inhibiting benastatins [57] and bequinostatins [72].

To provide an initial bioactivity assessment of frankiamicin A, we carried out assays of the compound against several bacterial, fungal, and protozoal strains; and cancer cell lines (Table 3). The results show no cytotoxic activity on the cancer cell lines examined, no inhibition of the fungus *C*. *albicans*, protozoan *T*. *cruzi*, or bacteria *S*. *pyogenes*, *A*. *baumanii*, *P*. *aeruginosa*, *or Y*. *pestis*. However, frankiamicin A did show weak antimicrobial activity against both wild-type and methicillin-resistant *S*. *aureus* (MRSA). While frankiamicin A does not show potent antimicrobial or anticancer activities, the limited scope of the strains and cell lines tested, and the assays employed, leaves open the possibility that frankiamicin A has more potent bioactivity in other assays. The results of our initial bioactivity tests of frankiamicin A are important for expanding our knowledge of structure-activity relationships among pentangular class compounds that can guide future structural modification using chemical, enzymatic, or genetic means.

**Table 3:** Frankiamicin A bioactivity assay results.

**Test strain/cell line**	**IC**_50_ **(μM)**	**MIC (μM)** ^[^ ^a^ ^]^	**MIC (μM)** ^[^ ^b^ ^]^
HeLa	>100	---	---
MCF7	>100	---	---
Jurkat	>100	---	---
*C*. *albicans*	>100	---	---
*T*. *cruzi*	>100	---	---
*S*. *pyogenes*	---	>100	>100
*A*. *baumanii*	---	>100	>100
*P*. *aeruginosa*	---	>100	>100
*Y*. *pestis*	---	>100	>100
*S*. *aureus*	---	∼100	>100
MRSA	---	∼50	>100

**[a]** Assessed after 6 h incubation.

**[b]** Assessed after 18 h incubation.

## Conclusions

The advent of high-throughput, low-cost bacterial genome sequencing is bringing to light thousands of unstudied natural product biosynthetic gene clusters from diverse and unexploited branches of the tree of life—so many that they cannot all be studied using traditional experimental approaches. Global bioinformatic and comparative genomic analysis facilitates more complete and integrated use of this large volume of sequence data, together with the existing experimentally-derived knowledge base, to select for experimental characterization specific gene clusters with atypical sequence characteristics. The results of such bioinformatics-guided characterization endeavors provide key links between gene clusters and the molecules they produce that can lead to a more detailed understanding of gene cluster sequence/function relationships within an entire class of natural products; and can serve as a solid foundation for generating additional biosynthetic hypotheses.

Here we applied such a global bioinformatic/comparative genomic approach to bacterial type II polyketide gene clusters. We identified within a subset of these clusters a clade of unstudied *Frankia* KSα/β enzymes that possess divergent sequence characteristics. We proposed based on comparative analysis of the remaining biosynthetic enzymes within these gene clusters that they biosynthesize a product with a pentangular or tetracenomycin core structure made from a poly-β-ketone intermediate of at least 24 carbons; and that the core structure undergoes minimal tailoring modifications. Identification, isolation, and structure elucidation of the compound produced by a representative of this class of gene clusters from *Frankia* sp. EAN1pec revealed that the cluster biosynthesizes the 24-carbon pentangular type II polyketide **4**, establishing the product specificity of the KSα/β and demonstrating the collective function of the cyclases. Further comparative analysis of *Frankia* cluster enzymes with homologues in training set pentangular clusters led us to suggest refined roles for conserved ABM superfamily members FkmO1 and FkmO2 and their homologues.

We believe that global bioinformatic/comparative genomic methods, such as those described here, can be an invaluable tool to guide experimental work aimed at expanding our understanding of natural product gene cluster sequence-function relationships; and for generating new biosynthetic hypotheses.

## Supporting Information

S1 Fig Schematic summary of *Dynamite* workflow used in this study. (PDF)

S2 Fig High resolution version of the ketosynthase α/β dendrogram shown in Fig. 3 with bootstrap values. (PDF)

S3 Fig ^1^H NMR spectrum of frankiamicin A (4) (PDF)

S4 Fig ^13^C NMR spectrum of frankiamicin A (4) (PDF)

S5 Fig ^1^H-^1^H COSY spectrum of frankiamicin A (4) (PDF)

S6 Fig HMQC spectrum of frankiamicin A (4) (PDF)

S7 Fig HMBC spectrum of frankiamicin A (4) (PDF)

S8 Fig Comparison of ^13^C spectra of unlabeled frankiamicin A (4) and frankiamicin A obtained by feeding [1,2-13C2]acetate doped with unlabeled compound. (PDF)

S1 Table List of ketosynthase α/β genes used to construct the dendrogram shown in Fig. 3; and their associated metadata. (PDF)

S2 Table List of type I polyketide, type II polyketide, and non-ribosomal peptide natural product gene clusters identified in *Frankia* genomes using *Dynamite* software. (PDF)

S3 Table Information on the *Frankia* genomes analyzed as part of this study. (PDF)

S4 Table Comparative genomic summary of *Frankia* type II polyketide gene cluster biosynthetic proteins and their homologues in pentangular and tetracenomycin training set clusters. (PDF)
