# Correction: Expanding our Understanding of Sequence-Function Relationships of Type II Polyketide Biosynthetic Gene Clusters: Bioinformatics-Guided Identification of Frankiamicin A from *Frankia* sp. EAN1pec

**DOI:** 10.1371/journal.pone.0129408

**Published:** 2015-06-01

**Authors:** Yasushi Ogasawara, Benjamin J. Yackley, Jacob A. Greenberg, Snezna Rogelj, Charles E. Melançon

The following information is missing from the Funding section: This work was supported by NIH NM-INBRE grant P20 GM103451 (CEM) and by start-up funds from University of New Mexico (CEM).

There are errors in the legend for Fig 1, “Structures of prototypical type II polyketides”. Please view Fig 1 and its complete, correct legend here.

**Fig 1:**
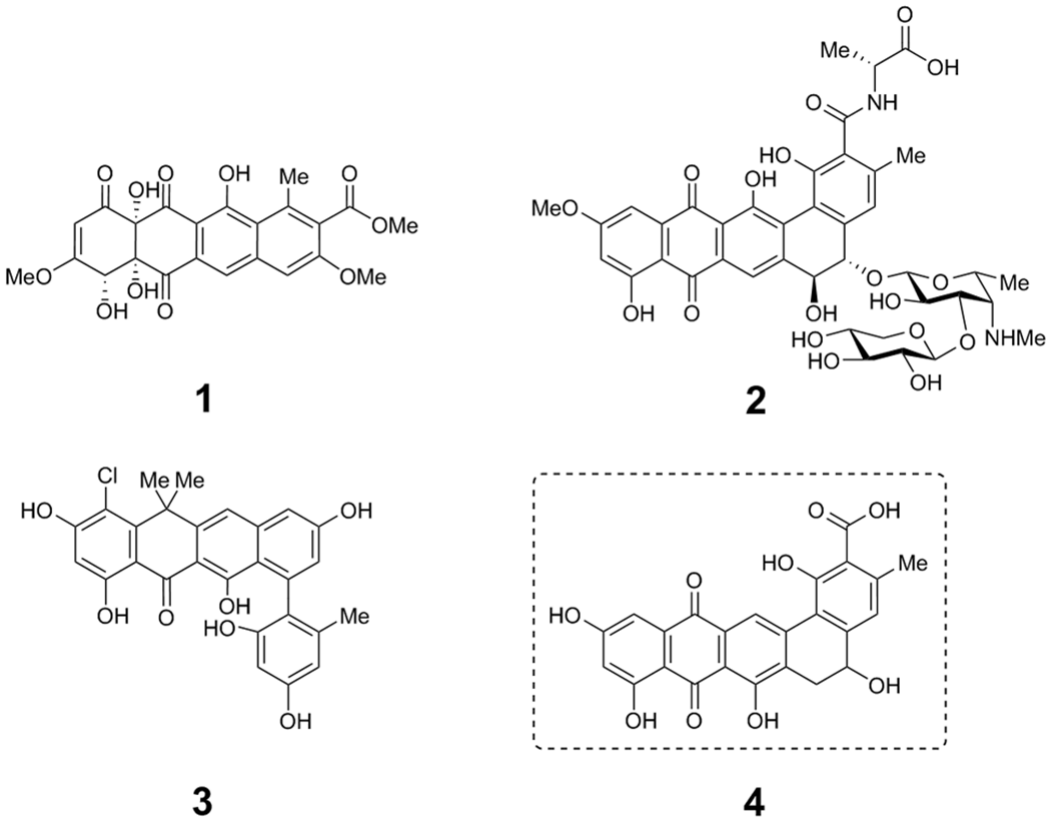
Structures of prototypical type II polyketides. Structures of tetracenomycin C (**1**), pradimicin A (**2**), fasamycin A (**3**), and the pentangular polyketide frankiamicin A (**4**) identified in this study.
