# Supplementary figures and images for: Expanding our Understanding of Sequence-Function Relationships of Type II Polyketide Biosynthetic Gene Clusters: Bioinformatics-Guided Identification of Frankiamicin A from Frankia sp. EAN1pec
Source: PLoS One. 2015 Apr 2;10(4):e0121505. doi: 10.1371/journal.pone.0121505 (PMC4383371; doi:10.1371/journal.pone.0121505)

Figure S1. Schematic summary of *Dynamite* workflow used in this study.

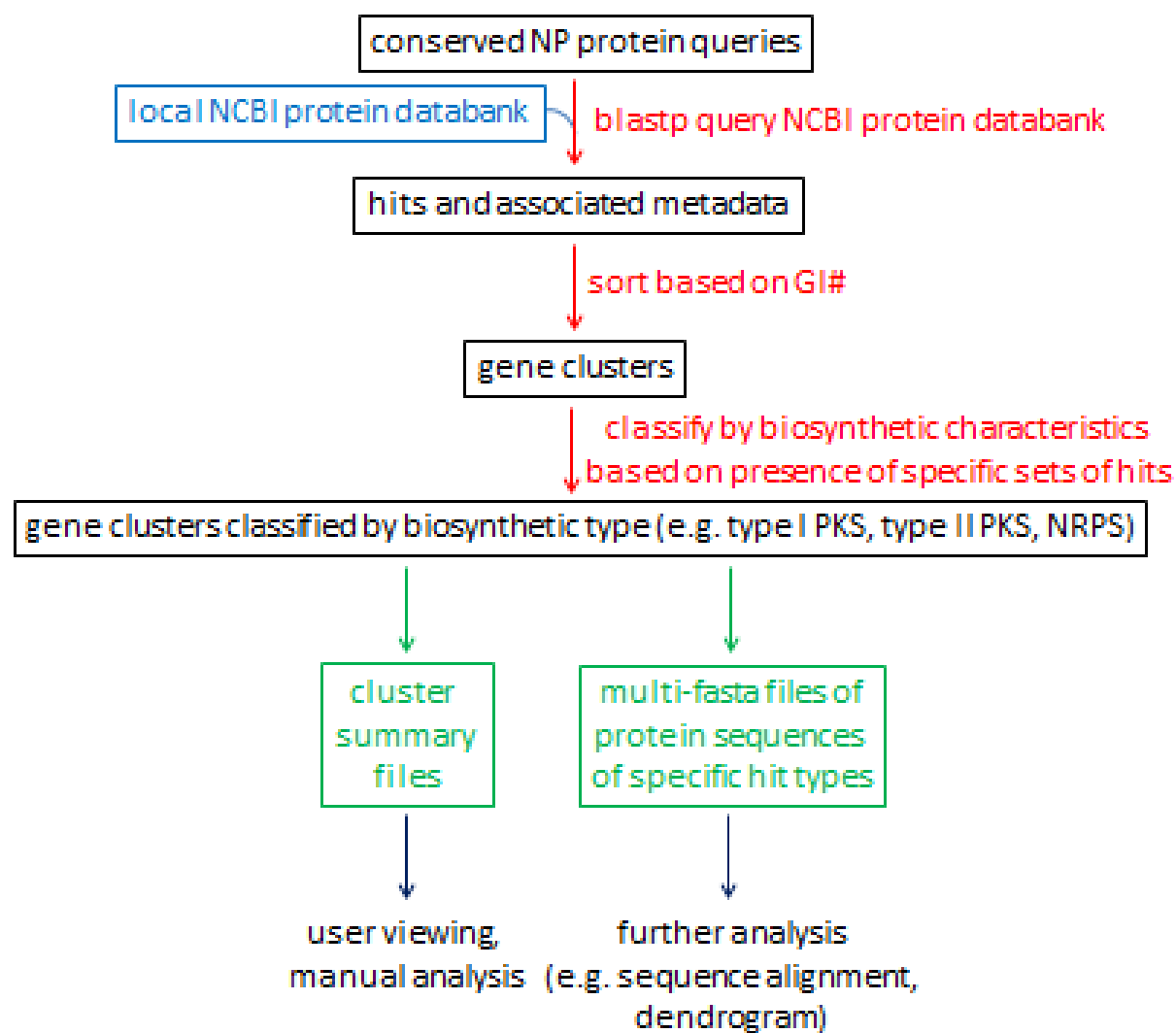

Supplement: S1 Fig — (PDF) [file pone.0121505.s001.pdf]

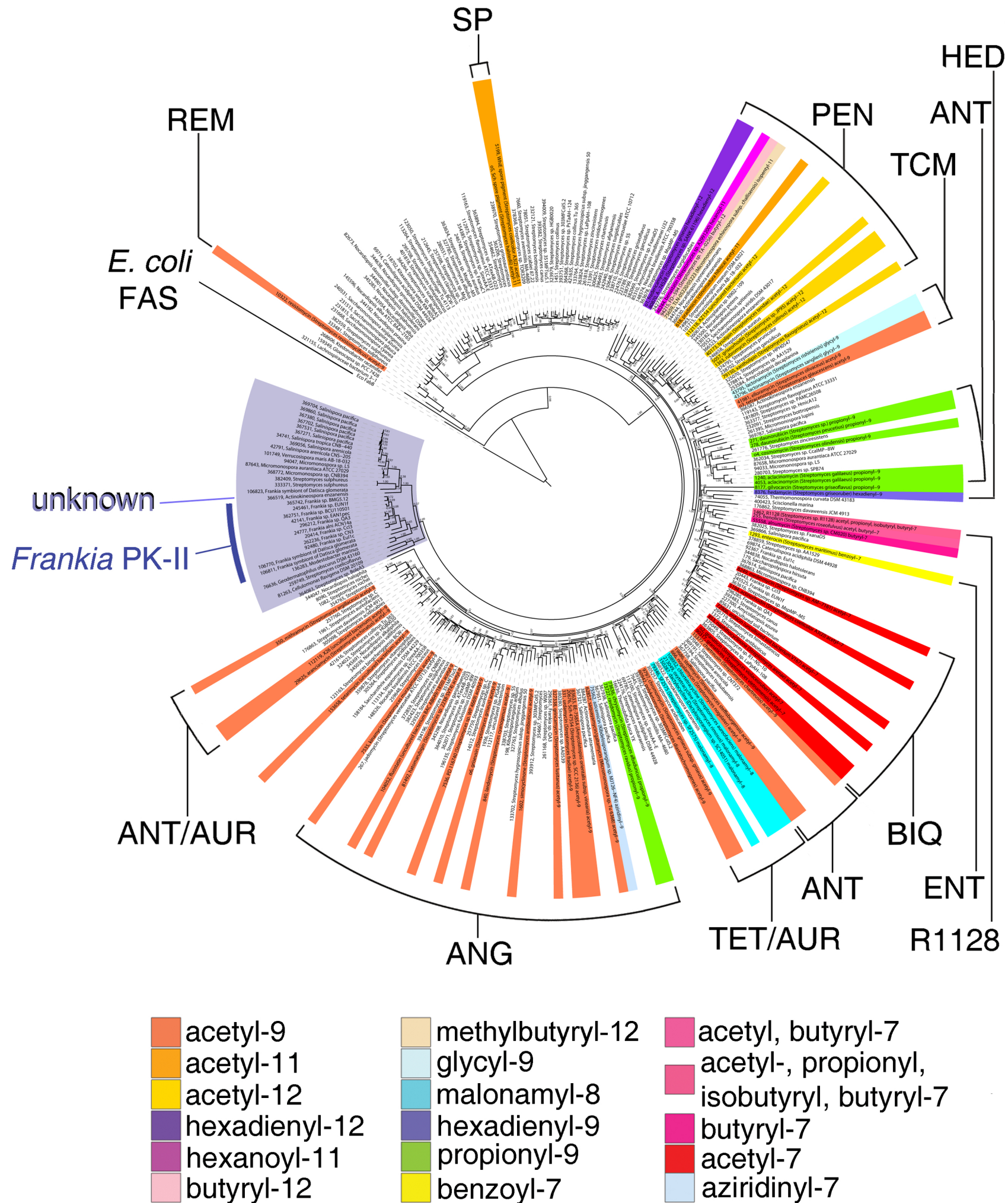

Supplement: S2 Fig — (PDF) [file pone.0121505.s002.pdf]

Frankiamicin A,  $^1\text{H}$  NMR, 500 MHz,  $\text{DMSO}-d_6$

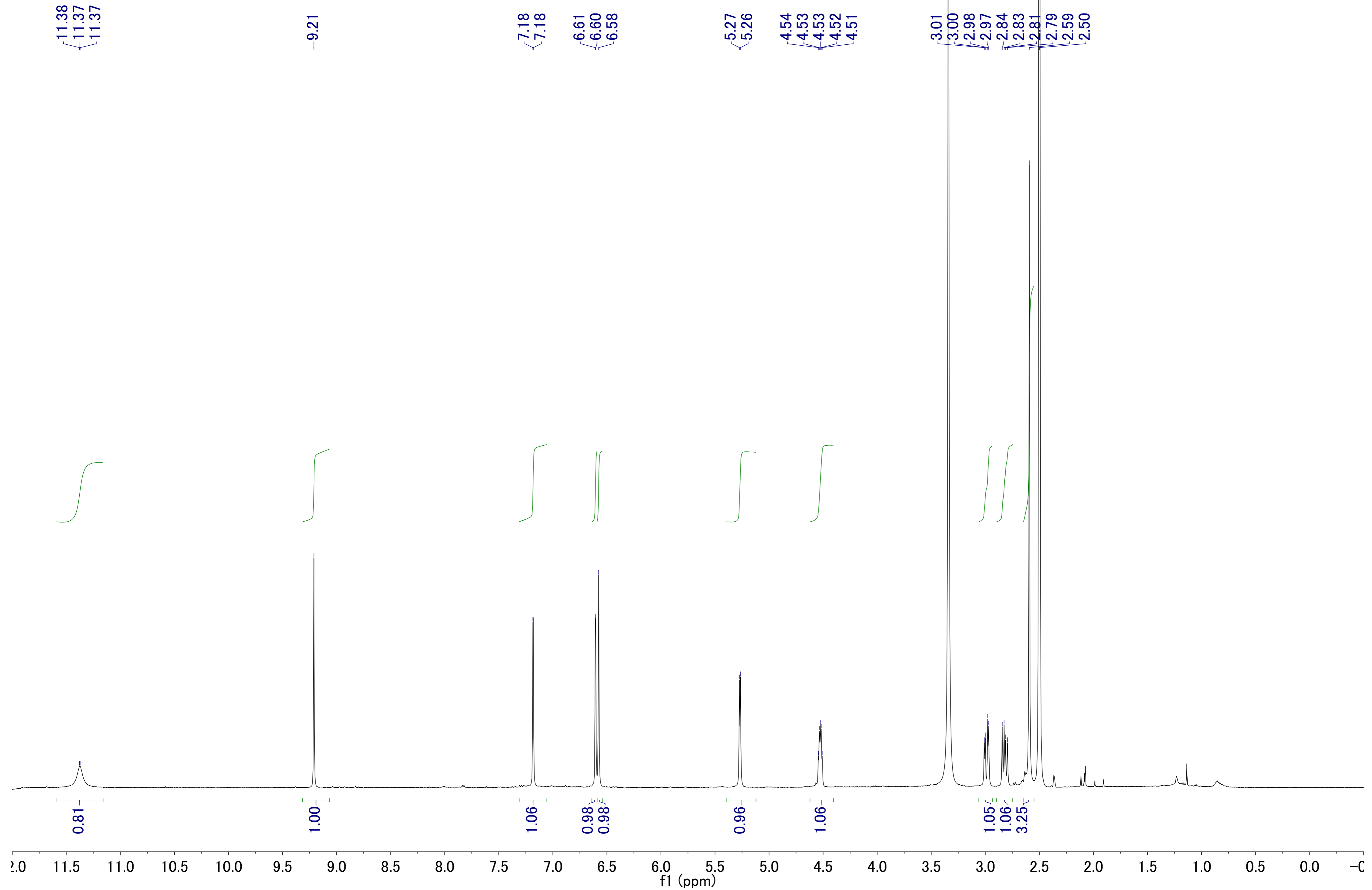

Supplement: S3 Fig — (PDF) [file pone.0121505.s003.pdf]

Frankiamicin A,  $^{13}\text{C}$  NMR, 75 MHz,  $\text{DMSO}-d_6$

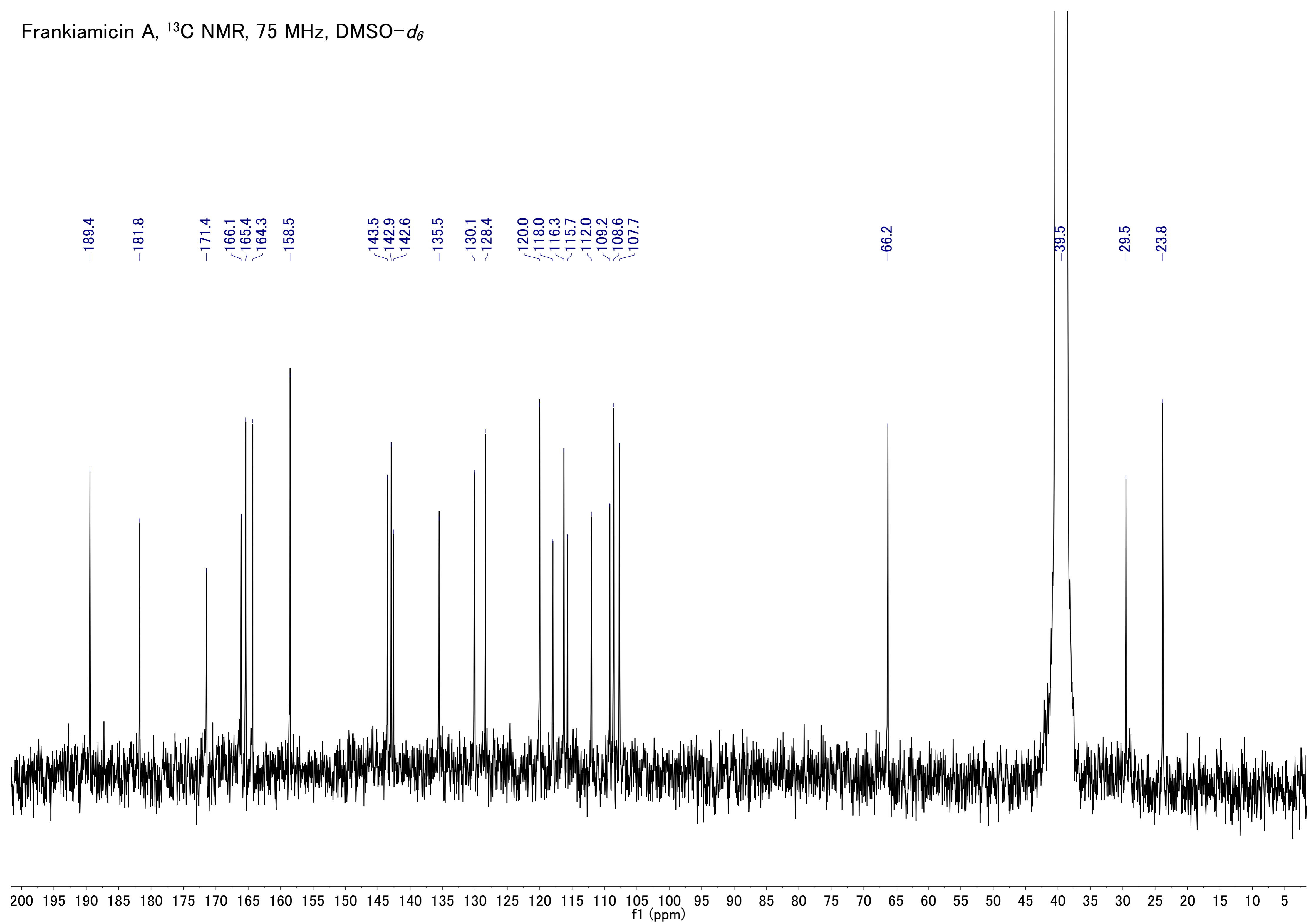

Supplement: S4 Fig — (PDF) [file pone.0121505.s004.pdf]

Frankiamicin A,  $^1\text{H}$ - $^1\text{H}$  COSY, 500 MHz,  $\text{DMSO}-d_6$

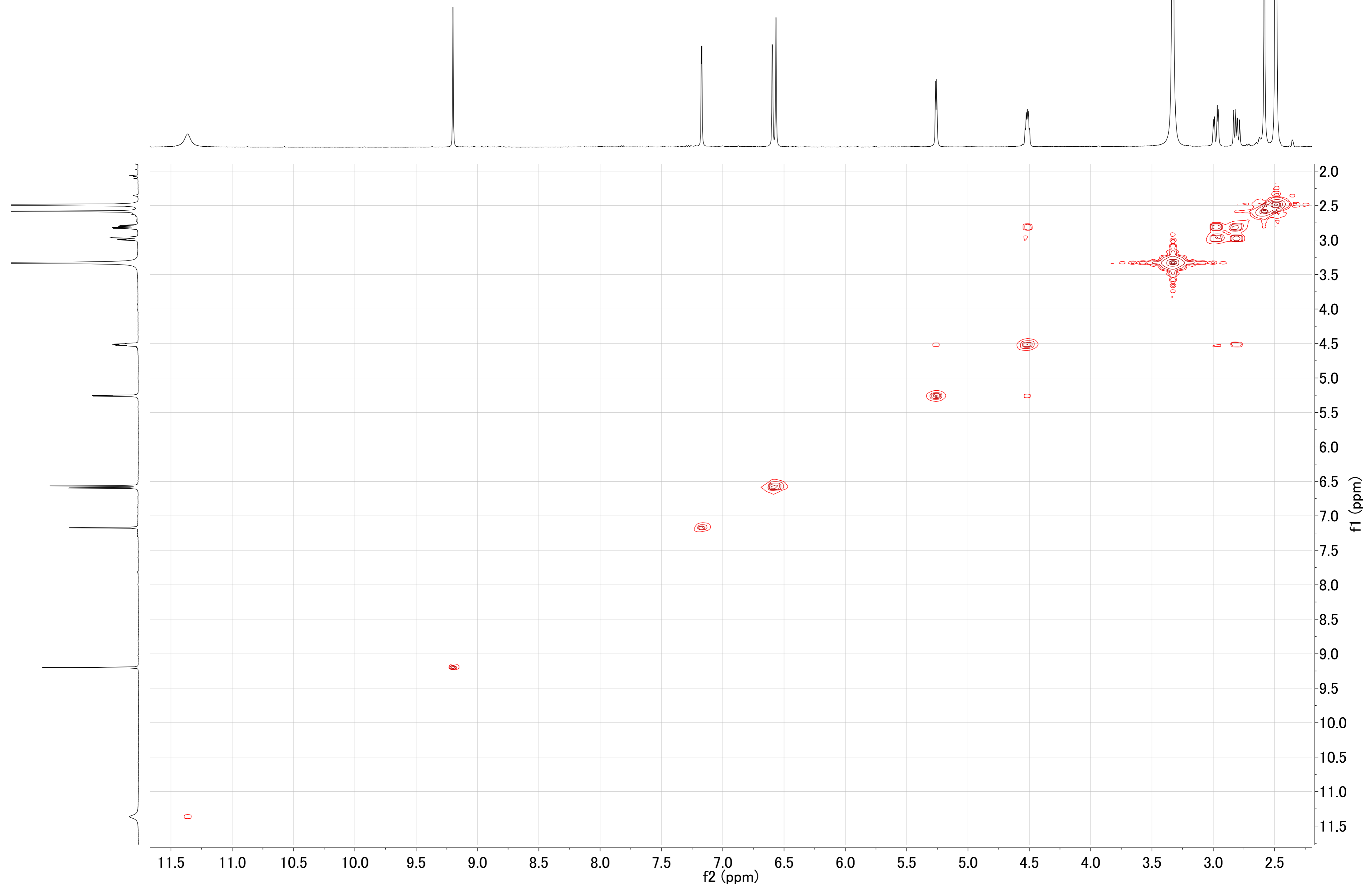

Supplement: S5 Fig — (PDF) [file pone.0121505.s005.pdf]

Frankiamicin A, HMQC, 500 MHz, DMSO- $d_6$

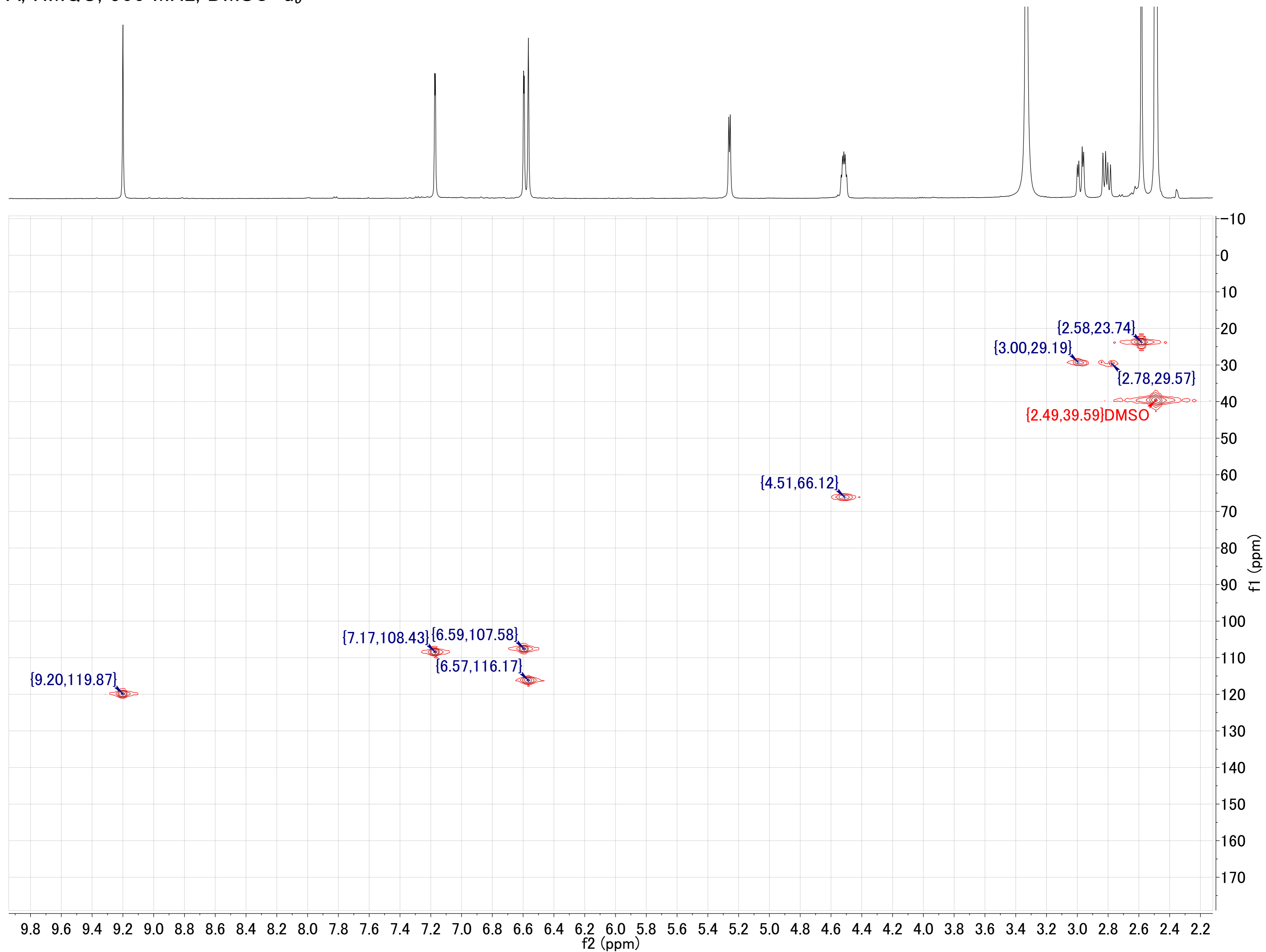

Supplement: S6 Fig — (PDF) [file pone.0121505.s006.pdf]

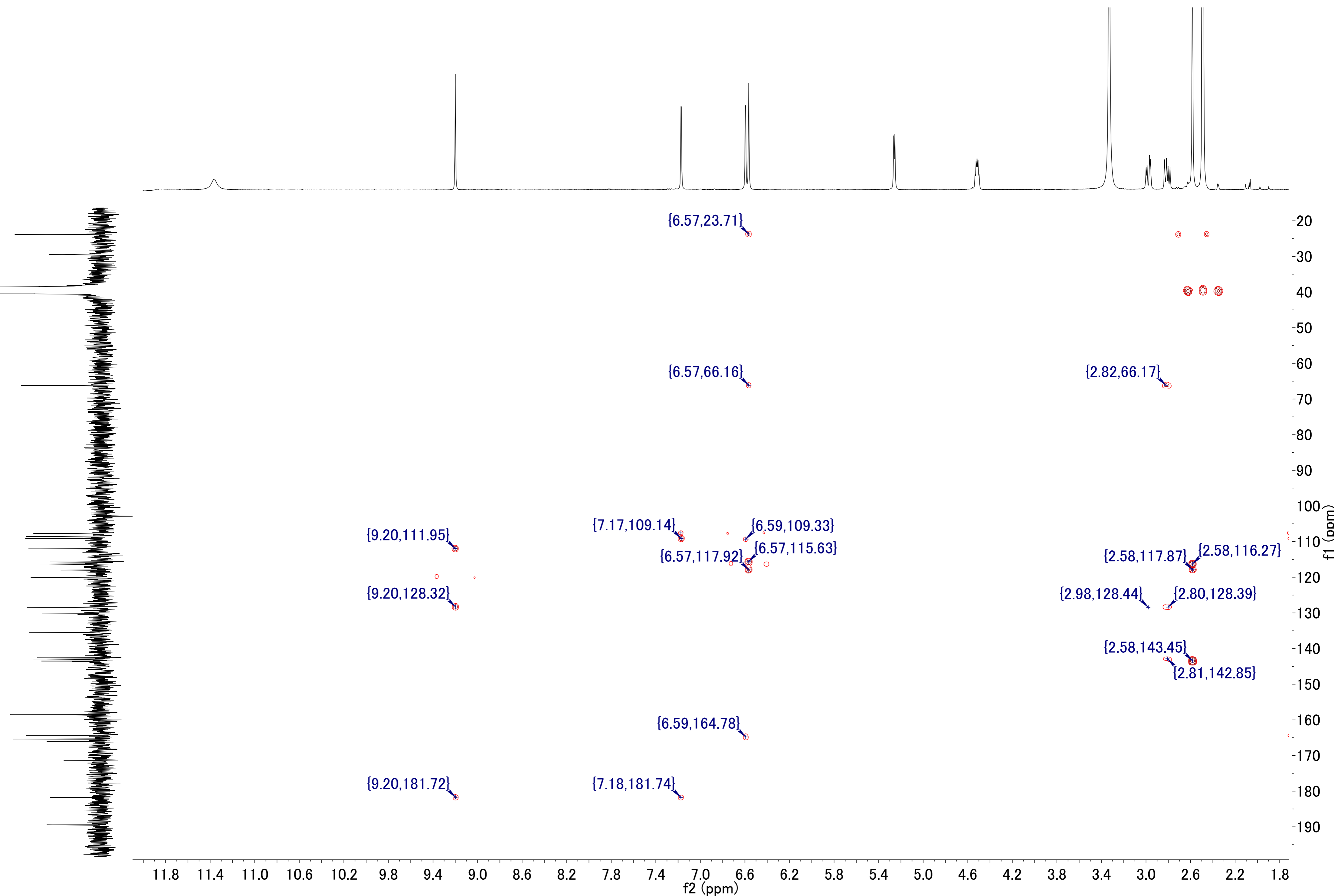

Supplement: S7 Fig — (PDF) [file pone.0121505.s007.pdf]
