# Supplementary material for: Expanding our Understanding of Sequence-Function Relationships of Type II Polyketide Biosynthetic Gene Clusters: Bioinformatics-Guided Identification of Frankiamicin A from Frankia sp. EAN1pec
Source: PLoS One. 2015 Apr 2;10(4):e0121505. doi: 10.1371/journal.pone.0121505 (PMC4383371; doi:10.1371/journal.pone.0121505)
Supplement: S8 Fig — (PDF) [file pone.0121505.s008.pdf]

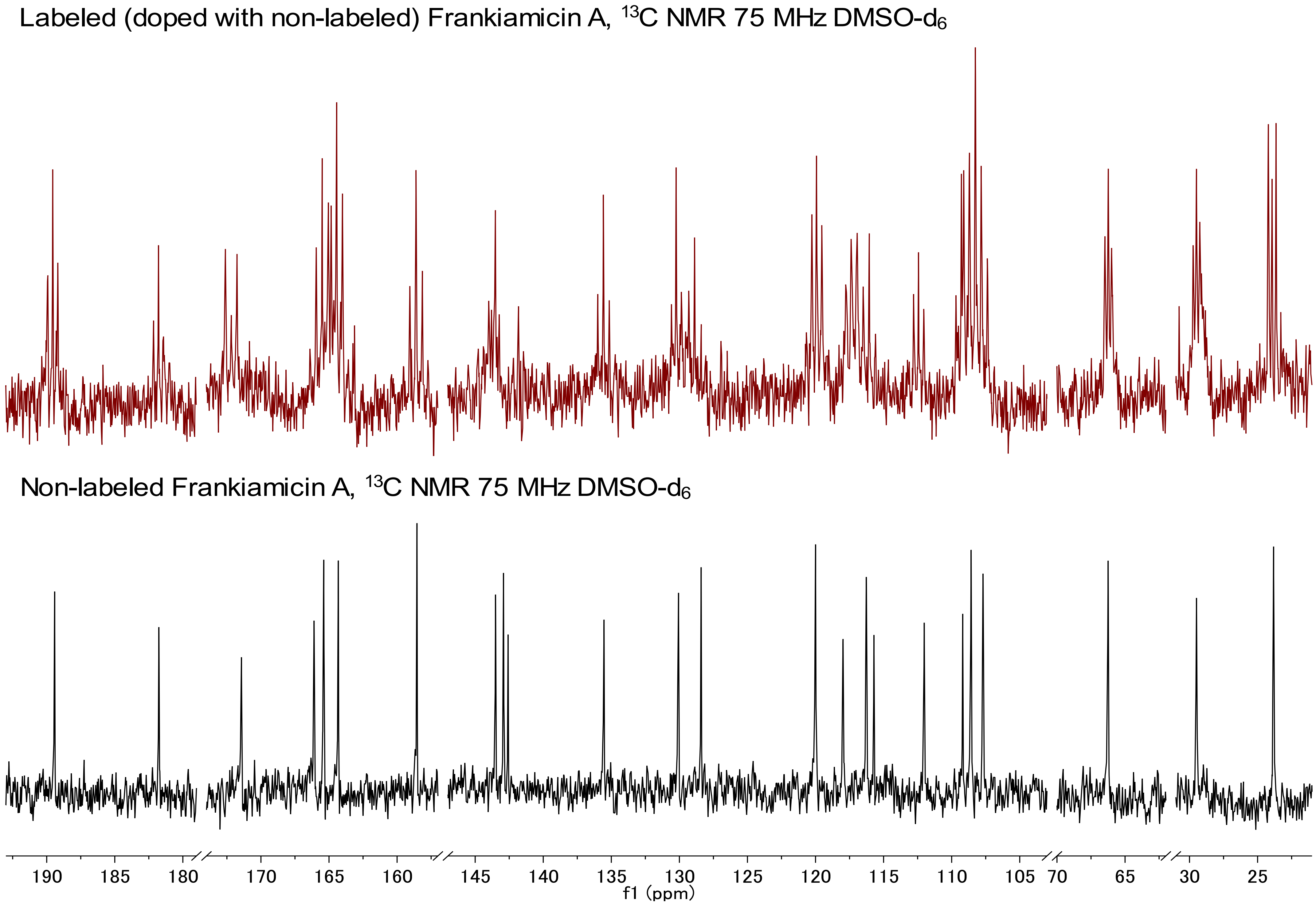
Labeled (doped with non-labeled) Frankiamicin A, ^{13}C NMR 75 MHz DMSO-d_6
Non-labeled Frankiamicin A, ^{13}C NMR 75 MHz DMSO-d_6
190 185 180 170 165 160 145 140 135 130 125 120 115 110 105 70 65 30 25
f1 (ppm)
