# Supplementary material for: Expanding our Understanding of Sequence-Function Relationships of Type II Polyketide Biosynthetic Gene Clusters: Bioinformatics-Guided Identification of Frankiamicin A from Frankia sp. EAN1pec
Source: PLoS One. 2015 Apr 2;10(4):e0121505. doi: 10.1371/journal.pone.0121505 (PMC4383371; doi:10.1371/journal.pone.0121505)
Supplement: S1 Table — (PDF) [file pone.0121505.s009.pdf]

Table S1. List of ketosynthase α/β genes used to construct the dendrogram shown in Figure 3; and associated metadata.

PKS_Cluster_ID	Strain name	Compound name	starter unit - number of cycles	NCBI gi Ksα	NCBI gi KSβ gi
179	Saccharopolyspora hirsuta			347180	347181
198	Kibdelosporangium aridum			406081	406082
255	Streptomyces roseofulvus	frenolicin	acetyl, butyryl-7	487889	487890
267	Streptomyces venezuelae ATCC 10712	jadomycin	acetyl-9	510722	510723
271	Streptomyces sp.	daunorubicin	propionyl-9	516109	516110
278	Streptomyces peucetius	daunorubicin	propionyl-9	532245	532246
331	Streptomyces fradiae	urdamycin	acetyl-9	809105	809106
350	Streptomyces argillaceus	mithramycin	acetyl-9	927517	927518
618	Actinomadura hibisca	pradimicin	acetyl-11	2580442	2580443
666	Streptomyces nogalater	nogalamycin	acetyl-9	2916812	2916813
840	Streptomyces cyanogenus	landomycin	acetyl-9	4240405	4240406
853	Streptomyces arenae	naphthocyclinone	acetyl-7	4416222	4416223
1082	Streptomyces rochei			6518511	6518512
1240	Streptomyces galilaeus	aclacinomycin	propionyl-9	7800665	7800666
1293	Streptomyces maritimus	enterocin	benzoyl-7	8926190	8926191
1363	Streptomyces collinus	rubromycin	acetyl-12	9944994	9944995
1372	Streptomyces antibioticus			9967595	9967596
1451	Streptomyces collinus			11024335	11024336
1462	Streptomyces sp. R1128	R1128	acetyl, propionyl, isobutyryl, butyryl-7	11096114	11096113
1602	Streptomyces antibioticus	simocyclinone	acetyl-9	12744820	12744821
1936	Streptomyces sp. PGA64			14280343	14280344
1961	Streptomyces aureofaciens			14486277	14486278
4053	Streptomyces galilaeus	aclacinomycin	propionyl-9	16945714	16945715
5057	Streptomyces sp. JP95	griseorhodin	acetyl-12	21039488	21039489
5194	Streptomyces coelicolor A3(2)	actinorhodin	acetyl-7	21223458	21223459
5199	Streptomyces coelicolor A3(2)	WhiE spore pigment	acetyl-11	21223681	21223680
7535	Streptomyces murayamaensis	kinamycin	acetyl-9	29469233	29469234
7536	Streptomyces sp. WP 4669	PD 116740	acetyl-9	29469252	29469253
7645	Streptomyces avermitilis MA-4680			29828918	29828917
7660	Streptomyces avermitilis MA-4680			29829380	29829381
8090	Streptomyces rochei			30795041	30795040
8177	Streptomyces griseoflavus	gilvocarcin	propionyl-9	32140283	32140284
8249	Streptomyces sp. AM-7161	medermycin	acetyl-7	32469270	32469271
8376	Streptomyces griseoruber	hedamycin	hexadienyl-9	32492544	32492543
9797	Streptomyces griseus subsp. griseus	chromomycin	acetyl-9	40644834	40644833
10322	Streptomyces resistomycificus	resistomycin	acetyl-9	45259316	45259317
14512	Streptomyces aureofaciens			61968692	61968693
15377	Streptomyces chartreusis	chartreusin	acetyl-9	68146474	68146475
16636	Thermobifida fusca YX			72161622	72161623
19686	Streptomyces steffisburgensis	steffimycin	acetyl-9	84619196	84619195
20414	Frankia sp. CcI3			86741538	86741537
20443	Frankia sp. CcI3			86742778	86742777
20676	Streptomyces sp. SCC 2136	Sch 47554	acetyl-9	88319793	88319792
24777	Frankia alni ACN14a			111223784	111223783
29025	Streptomyces echinatus	aranciamycin	acetyl-9	118722503	118722502
34741	Salinispora tropica CNB-440			145595027	145595026
40173	Streptomyces tendae	lysolipin	acetyl-12	154623217	154623216
41981	Streptomyces olivaceus	elloramycin	acetyl-9	158148282	158148283
42141	Frankia sp. EAN1pec			158314223	158314224
42791	Salinispora arenicola CNS-205			159038259	159038258
43795	Streptomyces rishiriensis	lactonamycin	glycyl-9	161367388	161367389
43796	Streptomyces sanglieri	lactonamycin	glycyl-9	161367423	161367424
46244	Streptomyces sp. A2991200	benastatin	hexanoyl-11	169402965	169402966
55558	Streptomyces sp. CM020	alnumycin	butyryl-7	209863916	209863917
60942	Streptomyces diastatochromogenes	polyketomycin	acetyl-9	224812396	224812397
62857	Micromonospora sp. Tu 6368	saquayamycin/galtamycin	acetyl-9	227121321	227121322
69714	Catenulispora acidiphila DSM 44928			256390289	256390290
69785	Catenulispora acidiphila DSM 44928			256392728	256392729
69874	Catenulispora acidiphila DSM 44928			256395612	256395613
70552	Saccharomonospora viridis DSM 43017			257057328	257057329
72906	uncultured soil bacterium V167	erdacin	acetyl-7	261497157	261497158
73636	Streptomyces albaduncus	chrysomycin	propionyl-9	266631088	266631089
73727	Streptomyces ravidus	ravidomycin	propionyl-9	268322287	268322286
74055	Thermomonospora curvata DSM 43183			269126987	269126986
74963	Streptosporangium roseum DSM 43021			271965601	271965602
75562	Kibdelosporangium sp. MJ126-NF4	azicemicin	aziridinyl-9	282801740	282801741
75659	Micromonospora echinospora subsp. challisensis	TLN-05220/TLN-05223	2-methylbutyryl-12	283484105	283484106
76636	Geodermatophilus obscurus DSM 43160			284988769	284988768
78051	Streptomyces scabiei 87.22			290958762	290958763
79150	Streptomyces flavogriseus	xantholipin	acetyl-12	292386134	292386133
79346	Streptomyces sp. SF2575	SF2575	malonamyl-8	292659136	292659137
80870	Streptomyces sp. SANK 61196	A-74528	hexadienyl-12	296046088	296046089
81263	Cellulomonas flavigena DSM 20109			296131162	296131163
81492	Streptomyces sp. 2238-SVT4	hatomarubigin	acetyl-9	296178419	296178421
82673	Nocardiopsis dassonvillei subsp. dassonvillei DSM			297563194	297563193
83633	Amycolatopsis orientalis subsp. vinearia	BE-7585A	acetyl-9	298256334	298256335
85310	Amycolatopsis mediterranei U32			300787306	300787305
85631	uncultured soil bacterium			301057030	301057029
87643	Micromonospora aurantiaca ATCC 27029			302867962	302867961
87658	Micromonospora aurantiaca ATCC 27029			302868481	302868480
90317	Streptomyces vietnamensis	granaticins	acetyl-7	308445212	308445213
92367	Frankia sp. EuI1c			312195193	312195194
92480	Frankia sp. EuI1c			312198553	312198552
94033	Micromonospora sp. L5			315505143	315505144
94047	Micromonospora sp. L5			315505638	315505639
94215	Streptomyces sp. TA-0256	FD-594	butyryl-12	316997093	316997094

101715	Verrucosispora maris AB-18-032			330467594	330467593
101749	Verrucosispora maris AB-18-032			330468706	330468705
104052	uncultured bacterium BAC AB649/1850	fluostatin	acetyl-9	332380592	332380591
106770	Frankia symbiont of Datisca glomerata			336176581	336176580
106811	Frankia symbiont of Datisca glomerata			336178278	336178279
106823	Frankia symbiont of Datisca glomerata			336178651	336178650
108952	Streptomyces aureofaciens	chlortetracycline	malonamyl-8	338776764	338776763
112117	uncultured bacterium			343479049	343479050
112118	uncultured bacterium	AZ154	acetyl-12	343479100	343479099
112119	uncultured bacterium	X26	acetyl-9	343479142	343479141
112975	Streptomyces sp. SirexAA-E			344997887	344997886
113100	Streptomyces sp. SirexAA-E			345002705	345002706
113134	Streptomyces sp. SirexAA-E			345003803	345003804
113264	Streptomyces violaceusniger Tu 4113			345010189	345010188
118702	Kitasatospora setae KM-6054			357394114	357394115
119143	Streptomyces flavogriseus ATCC 33331			357414496	357414495
119163	Streptomyces flavogriseus ATCC 33331			357415186	357415187
123050	Streptomyces bingchenggensis BCW-1			374985521	374985522
123163	Streptomyces bingchenggensis BCW-1			374989600	374989601
133702	Streptomyces hygroscopicus subsp. jinggangensis 50			386837281	386837280
133764	Streptomyces hygroscopicus subsp. jinggangensis 50			386839769	386839768
136283	Modestobacter marinus			389861877	389861876
145596	Nocardiopsis alba ATCC BAA-2165			403508105	403508106
148526	Nocardia brasiliensis ATCC 700358			407642667	407642668
148574	Nocardia brasiliensis ATCC 700358			407644198	407644199
150287	Dactylosporangium sp. SC14051	dactylocycline	malonamyl-8	408451285	408451286
150648	Streptomyces venezuelae ATCC 10712			408682514	408682513
153658	uncultured bacterium	tetarimycin	acetyl-9	426272821	426272820
158184	Saccharothrix espanaensis DSM 44229			433607249	433607250
159349	Gloeocapsa sp. PCC 7428			434395464	434395463
176862	Streptomyces davawensis JCM 4913			471321947	471321946
176863	Streptomyces davawensis JCM 4913			471321991	471321990
181809	Streptomyces sp. PAMC26508			479318246	479318247
196135	Streptomyces fulvissimus DSM 40593			488611453	488611454
212645	Streptomyces clavuligerus			294328345	294328346
213049	Streptomyces rimosus	oxytetracycline	malonamyl-8	440620236	440620260
213391	Streptomyces viridochromogenes			302471833	302471834
223789	Streptomyces griseoflavus			302474694	302474695
224176	Streptomyces mobaraensis			453051297	453051296
224763	Streptomyces			291344118	291344117
227300	Amycolatopsis azurea			449420704	449420705
231513	Saccharomonospora azurea			359738726	359738725
231668	Saccharomonospora cyanea			374661362	374661363
231815	Saccharomonospora glauca			384521847	384521848
232091	Streptomyces bottropensis			456387833	456387834
232121	Streptomyces bottropensis			456387348	456387347
238702	Streptomyces gancidicus			455651441	455651440
238799	Streptomyces gancidicus			455647826	455647827
238970	Streptomyces griseoaurantiacus			329303135	329303134
240531	Saccharomonospora xinjiangensis			383464465	383464464
242194	Streptomyces tsukubaensis			385668968	385668967
242776	Streptomyces turgidiscabies			440283088	440283085
242940	Streptomyces turgidiscabies			440276217	440276216
245461	Frankia sp. EUN1f			288352379	288352380
245525	Frankia sp. EUN1f			288350346	288350347
246658	Streptomyces auratus			396997410	396997409
246748	Streptomyces auratus			396993109	396993110
253584	Amycolatopsis decaplanina			452952207	452952208
257598	Streptomyces sp. C			302441586	302441587
257759	Streptomyces sp. C			302448244	302448245
257760	Streptomyces sp. C			302448272	302448273
259648	Streptomyces sviceus			197711929	197711928
259749	Streptomyces coelicoflavus			371551784	371551785
260674	Actinoplanes sp. N902-109			494685525	494685524
261168	Streptomyces sp. W007			364006457	364006458
261395	Micromonospora lupini			385884259	385884258
261776	Streptomyces zinciresistens			345639059	345639058
261818	Streptomyces zinciresistens			345637421	345637420
262236	Frankia sp. CN3			357077380	357077379
268606	Ktedonobacter racemifer			297547788	297547789
280551	Streptomyces sp. Mg1			194344319	194344318
280703	Streptomyces sp. SPB74			197695599	197695598
295708	Streptomyces himastatinicus			302459209	302459210
295719	Streptomyces himastatinicus			302459575	302459574
296212	Frankia sp. QA3			392285106	392285107
296285	Frankia sp. QA3			392287527	392287528
296361	Frankia sp. QA3			392290229	392290230
300625	Streptomyces chartreusis			497734383	497734384
305098	Streptomyces acidiscabies			498039595	498039594
305257	Streptomyces acidiscabies			498045513	498045514
305264	Streptomyces acidiscabies			498045777	498045775
321155	Lachnospiraceae bacterium 3-1			507762612	507762611
323979	Streptomyces sp. HGB0020			512062730	512062729
324023	Streptomyces sp. HGB0020			512060372	512060371
325070	Streptomyces sp. HPH0547			512153434	512153433
327338	Streptomyces lusitanus	grincamycin	acetyl-9	514389165	514389166
327763	Streptomyces albulus			508092873	508092874
327785	Streptomyces albulus			508092166	508092165
329325	Streptomyces aurantiacus			514332066	514332067
330182	Actinoalloteichus spitiensis			515067606	515067605
333371	Streptomyces sulphureus			515467828	515467830

333481	Streptomyces sulphureus			515471814	515471813
338703	Streptomyces sp. SS			515806529	515806528
338710	Streptomyces sp. SS			515806720	515806721
343954	Nocardiopsis alba			516103130	516103129
344047	Nocardiopsis halophila			516106461	516106460
344192	Nocardiopsis prasina			516112287	516112286
344638	Nocardiopsis synnemataformans			516133090	516133089
344744	Nocardiopsis synnemataformans			516136652	516136651
344838	Nocardiopsis halotolerans			516140432	516140433
344901	Nocardiopsis halotolerans			516143136	516143135
345039	Nocardiopsis valliformis			516148637	516148638
345285	Nocardiopsis ganjiahuensis			516162729	516162731
345298	Nocardiopsis ganjiahuensis			516163860	516163858
345500	Nocardiopsis potens			516177487	516177485
345691	Nocardiopsis alkaliphila			516194141	516194140
354385	Streptomyces sp. FxanaC1			516769011	516769013
354662	Streptomyces			516790530	516790531
354667	Streptomyces			516790775	516790772
354765	Streptomyces			516797526	516797524
359878	Streptomyces vitaminophilus			517194301	517194300
362034	Streptomyces sp. CcalMP-8W			517298639	517298640
362071	Streptomyces sp. CcalMP-8W			517300121	517300122
362751	Frankia sp. BCU110501			517330197	517330198
363206	Streptomyces			517349015	517349014
363377	Streptomyces sp. HmicA12			517356095	517356094
363616	Streptomyces sp. MspMP-M5			517364723	517364724
363634	Streptomyces sp. MspMP-M5			517365652	517365653
363678	Streptomyces sp. MspMP-M5			517367650	517367649
363805	Streptomyces sp. LaPpAH-108			517372962	517372963
363824	Streptomyces sp. LaPpAH-108			517373726	517373725
363894	Streptomyces sp. ATexAB-D23			517376190	517376191
364010	Streptomyces sp. ATexAB-D23			517380234	517380233
364083	Streptomyces sp. BoleA5			517382660	517382661
364264	Streptomyces sp. BoleA5			517389779	517389778
364368	Streptomyces sp. PsTaAH-124			517393575	517393576
365742	Frankia sp. BMG5.12			517467538	517467539
366519	Actinokineospora enzanensis			517511338	517511339
366522	Actinokineospora enzanensis			517511425	517511424
366523	Actinokineospora enzanensis			517511485	517511486
366530	Actinokineospora enzanensis			517511634	517511633
366587	Actinokineospora enzanensis			517513802	517513801
367271	Salinispora pacifica			517549947	517549948
367321	Salinispora pacifica			517552055	517552054
367380	Salinispora pacifica			517554571	517554572
367531	Salinispora pacifica			517560266	517560265
367614	Salinispora pacifica			517563182	517563181
367702	Salinispora pacifica			517566341	517566340
367758	Salinispora pacifica			517568116	517568115
367938	Salinispora pacifica			517574884	517574885
367951	Salinispora pacifica			517575473	517575472
368772	Micromonospora sp. CNB394			517613873	517613874
368863	Micromonospora sp. CNB394			517617890	517617891
369056	Salinispora arenicola			517624668	517624667
369191	Salinispora arenicola			517629449	517629450
369636	Salinispora pacifica			517644688	517644689
369704	Salinispora pacifica			517647314	517647315
369787	Salinispora pacifica			517650873	517650874
369860	Salinispora pacifica			517653383	517653382
369866	Salinispora pacifica			517653809	517653808
370481	Streptomyces sp. CNT372			517676353	517676354
372859	Streptomyces sp. CNB091			517789797	517789796
374795	Streptomyces prunicolor			517891591	517891590
375049	Streptomyces sp. R1-NS-10			517900262	517900263
378368	Streptomyces sp. TOR3209			518156679	518156680
378814	Streptomyces sp. AA1529			518188243	518188242
378859	Streptomyces sp. AA1529			518189500	518189499
380280	Streptomyces sp. AA0539			518262143	518262142
382025	Streptomyces sp. FxanaD5			518354660	518354659
382069	Streptomyces sp. FxanaD5			518356686	518356687
382409	Streptomyces sulphureus			518373928	518373929
382423	Streptomyces sulphureus			518374555	518374554
382559	Streptomyces sulphureus			518379252	518379251
384287	Actinomadura atramentaria			518464803	518464802
393483	Streptomyces canus			518960103	518960102
393582	Streptomyces canus			518963441	518963442
393751	Streptomyces sp. 303MFCol5.2			518969878	518969877
393912	Streptomyces sp. 303MFCol5.2			518975094	518975095
393952	Streptomyces sp. 303MFCol5.2			518976223	518976224
394009	Streptomyces sp. 351MFTsu5.1			518978505	518978504
394196	Streptomyces sp. 351MFTsu5.1			518985500	518985501
396493	Streptomyces afghaniensis			514936636	514936635
400423	Sciscionella marina			521986047	521986046
401818	Streptomyces scabrisporus			522042542	522042543
421616	Streptomyces collinus Tu 365			529225472	529225473
421635	Streptomyces collinus Tu 365			529226334	529226333
o1	Streptomyces griseus	fredericamycin	hexadienyl-12	33327096	33327097
o2	Streptomyces violaceoruber	granaticin	acetyl-7	4218564	4218565
o3	Streptomyces glaucescens	tetracenomycin	acetyl-9	153496	153497
o4	Streptomyces olindensis	cosmomycin	propionyl-9	83272129	83272131
o5	Streptomyces halstedii	Sch spore pigment	acetyl-11	153323	153324
o6	Streptomyces griseus	griseusin	acetyl-9	581665	581666
