# Supplementary material for: Expanding our Understanding of Sequence-Function Relationships of Type II Polyketide Biosynthetic Gene Clusters: Bioinformatics-Guided Identification of Frankiamicin A from Frankia sp. EAN1pec
Source: PLoS One. 2015 Apr 2;10(4):e0121505. doi: 10.1371/journal.pone.0121505 (PMC4383371; doi:10.1371/journal.pone.0121505)
Supplement: S2 Table — (PDF) [file pone.0121505.s010.pdf]

Table S2. List of type I polyketide, type II polyketide, and non-ribosomal peptide natural product gene clusters identified in *Frankia* genomes using *Dynamite* software. Type II polyketide clusters are in bold, and those within the diverged clade examined in this study are labelled with 3 asterix in the "cluster type" column.

Organism	Cluster #	Gene cluster protein ID range		Cluster type
		First protein	Last protein	
Frankia alni ACN14a	1	111219827	111219853	cis-AT_PKS-I
Frankia alni ACN14a	2	111220746	111220752	cis-AT_PKS-I
Frankia alni ACN14a	3	111220995	111221007	cis-AT_PKS-I
Frankia alni ACN14a	4	111221105	111221124	cis-AT_PKS-I
Frankia alni ACN14a	5	111221959	111221990	cis-AT_PKS-I NRPS
Frankia alni ACN14a	6	111222328	111222337	cis-AT_PKS-I
Frankia alni ACN14a	7	111222397	111222407	cis-AT_PKS-I
Frankia alni ACN14a	8	111222599	111222615	cis-AT_PKS-I
Frankia alni ACN14a	9	111222836	111222874	cis-AT_PKS-I
Frankia alni ACN14a	10	111223473	111223488	cis-AT_PKS-I
Frankia alni ACN14a	11	111223558	111223561	NRPS
Frankia alni ACN14a	12	111223568	111223571	NRPS
**Frankia alni ACN14a**	**13**	**111223775**	**111223796**	**PKS-II *** **
Frankia sp. BCU110501	1	517315950	517315964	cis-AT_PKS-I
Frankia sp. BCU110501	2	517318311	517318315	cis-AT_PKS-I
Frankia sp. BCU110501	3	517318392	517318395	cis-AT_PKS-I
Frankia sp. BCU110501	4	517320080	517320097	cis-AT_PKS-I
Frankia sp. BCU110501	5	517321349	517321351	cis-AT_PKS-I
Frankia sp. BCU110501	6	517321360	517321360	trans-AT_PKS-I
Frankia sp. BCU110501	7	517321369	517321369	cis-AT_PKS-I
Frankia sp. BCU110501	8	517321380	517321416	cis-AT_PKS-I
Frankia sp. BCU110501	9	517321456	517321482	cis-AT_PKS-I
Frankia sp. BCU110501	10	517322903	517322915	cis-AT_PKS-I
Frankia sp. BCU110501	11	517326987	517327012	cis-AT_PKS-I
Frankia sp. BCU110501	12	517327259	517327270	cis-AT_PKS-I
Frankia sp. BCU110501	13	517327292	517327301	cis-AT_PKS-I
Frankia sp. BCU110501	14	517329256	517329256	NRPS
Frankia sp. BCU110501	15	517329264	517329282	cis-AT_PKS-I
**Frankia sp. BCU110501**	**16**	**517330195**	**517330199**	**PKS-II *** **
Frankia sp. BCU110501	17	517330237	517330238	cis-AT_PKS-I
Frankia sp. BCU110501	18	517330542	517330555	cis-AT_PKS-I
Frankia sp. BCU110501	19	517330874	517330877	cis-AT_PKS-I
Frankia sp. BCU110501	20	522061021	522061026	cis-AT_PKS-I
Frankia sp. BCU110501	21	522061077	522061102	cis-AT_PKS-I
Frankia sp. BCU110501	22	522061305	522061310	cis-AT_PKS-I
Frankia sp. BCU110501	23	522061453	522061455	NRPS
Frankia sp. BMG5.12	1	517464459	517464497	cis-AT_PKS-I
Frankia sp. BMG5.12	2	517464551	517464551	cis-AT_PKS-I
Frankia sp. BMG5.12	3	517464741	517464750	trans-AT_PKS-I
Frankia sp. BMG5.12	4	517466209	517466232	cis-AT_PKS-I
Frankia sp. BMG5.12	5	517466408	517466427	cis-AT_PKS-I
Frankia sp. BMG5.12	6	517467058	517467067	NRPS
**Frankia sp. BMG5.12**	**7**	**517467530**	**517467551**	**PKS-II *** **
Frankia sp. BMG5.12	8	517467647	517467666	cis-AT_PKS-I
Frankia sp. BMG5.12	9	517468621	517468636	cis-AT_PKS-I

Frankia sp. BMG5.12	10	517468646	517468661	cis-AT_PKS-I
Frankia sp. BMG5.12	11	517468751	517468764	cis-AT_PKS-I
Frankia sp. BMG5.12	12	517468895	517468903	NRPS
Frankia sp. BMG5.12	13	517469047	517469051	cis-AT_PKS-I
Frankia sp. BMG5.12	14	517469097	517469112	cis-AT_PKS-I
Frankia sp. BMG5.12	15	517469399	517469441	cis-AT_PKS-I
Frankia sp. BMG5.12	16	517469491	517469491	cis-AT_PKS-I
Frankia sp. BMG5.12	17	517469742	517469761	cis-AT_PKS-I
Frankia sp. BMG5.12	18	517469815	517469815	cis-AT_PKS-I
Frankia sp. CcI3	1	86566498	86566505	cis-AT_PKS-I
Frankia sp. CcI3	2	86566562	86566566	cis-AT_PKS-I
Frankia sp. CcI3	3	86567541	86567543	cis-AT_PKS-I NRPS
Frankia sp. CcI3	4	86568008	86568019	NRPS
Frankia sp. CcI3	5	86568473	86568480	cis-AT_PKS-I
Frankia sp. CcI3	6	86568529	86568538	cis-AT_PKS-I
Frankia sp. CcI3	7	86739636	86739643	cis-AT_PKS-I
Frankia sp. CcI3	8	86739700	86739704	cis-AT_PKS-I
Frankia sp. CcI3	9	86740679	86740681	cis-AT_PKS-I NRPS
Frankia sp. CcI3	10	86741146	86741157	NRPS
**Frankia sp. CcI3**	**11**	**86741535**	**86741560**	**PKS-II *****
Frankia sp. CcI3	12	86741611	86741618	cis-AT_PKS-I
Frankia sp. CcI3	13	86741667	86741676	cis-AT_PKS-I
**Frankia sp. CcI3**	**14**	**86742770**	**86742782**	**PKS-II**
Frankia sp. CN3	1	357070389	357070391	cis-AT_PKS-I
Frankia sp. CN3	2	357072034	357072039	cis-AT_PKS-I
Frankia sp. CN3	3	357076122	357076130	cis-AT_PKS-I
Frankia sp. CN3	4	357076290	357076301	cis-AT_PKS-I
**Frankia sp. CN3**	**5**	**357077366**	**357077423**	**PKS-II *****
Frankia sp. CN3	6	357077906	357077910	cis-AT_PKS-I
Frankia sp. CN3	7	357078885	357078885	NRPS
Frankia sp. CN3	8	357080303	357080323	NRPS
Frankia sp. CN3	9	357080338	357080369	NRPS
Frankia sp. CN3	10	357081620	357081629	cis-AT_PKS-I
**Frankia sp. EAN1pec**	**1**	**158314214**	**158314227**	**PKS-II *****
Frankia sp. EAN1pec	2	158314861	158314873	NRPS
Frankia sp. EAN1pec	3	158314895	158314896	cis-AT_PKS-I
Frankia sp. EAN1pec	4	158315159	158315199	cis-AT_PKS-I
Frankia sp. EAN1pec	5	158315264	158315282	cis-AT_PKS-I
Frankia sp. EAN1pec	6	158315408	158315418	cis-AT_PKS-I
Frankia sp. EAN1pec	7	158315653	158315678	cis-AT_PKS-I
Frankia sp. EAN1pec	8	158315715	158315756	cis-AT_PKS-I
Frankia sp. EAN1pec	9	158316046	158316050	cis-AT_PKS-I
Frankia sp. EAN1pec	10	158316591	158316614	cis-AT_PKS-I
Frankia sp. EAN1pec	11	158317359	158317367	cis-AT_PKS-I
Frankia sp. EAN1pec	12	158317499	158317526	cis-AT_PKS-I
Frankia sp. EAN1pec	13	158317682	158317684	NRPS
Frankia sp. EuI1c	1	312195087	312195107	cis-AT_PKS-I
**Frankia sp. EuI1c**	**2**	**312195185**	**312195226**	**PKS-II**
Frankia sp. EuI1c	3	312195425	312195429	cis-AT_PKS-I
Frankia sp. EuI1c	4	312196910	312196923	cis-AT_PKS-I
Frankia sp. EuI1c	5	312197187	312197202	cis-AT_PKS-I

Frankia sp. EuI1c	6	312198053	312198079	cis-AT_PKS-I
Frankia sp. EuI1c	7	312198185	312198244	cis-AT_PKS-I
Frankia sp. EuI1c	8	312198325	312198337	NRPS
**Frankia sp. EuI1c**	**9**	**312198547**	**312198562**	**PKS-II *** **
Frankia sp. EUN1f	1	288344572	288344577	cis-AT_PKS-I
Frankia sp. EUN1f	2	288344923	288344924	cis-AT_PKS-I
Frankia sp. EUN1f	3	288345064	288345067	cis-AT_PKS-I
Frankia sp. EUN1f	4	288345844	288345845	NRPS
Frankia sp. EUN1f	5	288346520	288346540	cis-AT_PKS-I
Frankia sp. EUN1f	6	288346877	288346877	cis-AT_PKS-I
Frankia sp. EUN1f	7	288346991	288346996	cis-AT_PKS-I
Frankia sp. EUN1f	8	288348482	288348511	cis-AT_PKS-I
Frankia sp. EUN1f	9	288349214	288349220	cis-AT_PKS-I
Frankia sp. EUN1f	10	288349375	288349390	cis-AT_PKS-I trans-AT_PKS-I
Frankia sp. EUN1f	11	288349805	288349811	trans-AT_PKS-I
Frankia sp. EUN1f	12	288349955	288349966	NRPS
Frankia sp. EUN1f	13	288350085	288350089	cis-AT_PKS-I
**Frankia sp. EUN1f**	**14**	**288350336**	**288350354**	**PKS-II**
**Frankia sp. EUN1f**	**15**	**288352340**	**288352390**	**PKS-II*** **
Frankia sp. QA3	1	392285059	392285062	NRPS
**Frankia sp. QA3**	**2**	**392285094**	**392285116**	**PKS-II *** **
Frankia sp. QA3	3	392285611	392285635	cis-AT_PKS-I
Frankia sp. QA3	4	392285841	392285872	cis-AT_PKS-I
Frankia sp. QA3	5	392286572	392286584	NRPS
**Frankia sp. QA3**	**6**	**392287513**	**392287543**	**PKS-II**
Frankia sp. QA3	7	392287695	392287706	cis-AT_PKS-I
Frankia sp. QA3	8	392287915	392287921	cis-AT_PKS-I
Frankia sp. QA3	9	392288746	392288770	cis-AT_PKS-I
**Frankia sp. QA3**	**10**	**392290227**	**392290257**	**PKS-II**
Frankia symbiont of Datisca glomerata	1	336176511	336176516	cis-AT_PKS-I
**Frankia symbiont of Datisca glomerata**	**2**	**336176578**	**336176596**	**PKS-II**
Frankia symbiont of Datisca glomerata	3	336177342	336177348	cis-AT_PKS-I
Frankia symbiont of Datisca glomerata	4	336177905	336177938	cis-AT_PKS-I
**Frankia symbiont of Datisca glomerata**	**5**	**336178258**	**336178279**	**PKS-II *** **
Frankia symbiont of Datisca glomerata	6	336178515	336178538	cis-AT_PKS-I
**Frankia symbiont of Datisca glomerata**	**7**	**336178649**	**336178662**	**PKS-II *** **
Frankia symbiont of Datisca glomerata	8	336178763	336178784	cis-AT_PKS-I
