# Supplementary material for: Expanding our Understanding of Sequence-Function Relationships of Type II Polyketide Biosynthetic Gene Clusters: Bioinformatics-Guided Identification of Frankiamicin A from Frankia sp. EAN1pec
Source: PLoS One. 2015 Apr 2;10(4):e0121505. doi: 10.1371/journal.pone.0121505 (PMC4383371; doi:10.1371/journal.pone.0121505)
Supplement: S3 Table — (PDF) [file pone.0121505.s011.pdf]

Table S3. Information on the *Frankia* genomes analyzed as part of this study.

Organism/Name	Accession #	WGS	# of Scaffolds	# of Genes	# of Proteins	Release Date	Status
Frankia alni ACN14a	NC_008278.1	-	1	6775	6700	8/3/2006	Complete
Frankia sp. BCU110501	-	ARDT01	194	6047	5880	4/19/2013	Scaffold
Frankia sp. BMG5.12	-	ARFH01	135	5473	5340	4/19/2013	Scaffold
Frankia sp. CcI3	NC_007777.1	-	1	4618	4499	2/6/2006	Complete
Frankia sp. CN3	-	AGJN02	2	7173	7024	11/15/2011	Scaffold
Frankia sp. EAN1pec	NC_009921.1	-	1	7377	7191	10/10/2007	Complete
Frankia sp. EuI1c	NC_014666.1	-	1	7263	7083	11/5/2010	Complete
Frankia sp. EUN1f	-	ADGX01	396	8236	8182	2/4/2010	Contig
Frankia sp. QA3	NZ_CM001489.1	AJWA01	1	6546	6033	5/8/2012	Chromosome
Frankia symbiont of *Datisca glomerata*	NC_015656.1	-	3	4597	4215	6/6/2011	Complete
