# Supplementary material for: Expanding our Understanding of Sequence-Function Relationships of Type II Polyketide Biosynthetic Gene Clusters: Bioinformatics-Guided Identification of Frankiamicin A from Frankia sp. EAN1pec
Source: PLoS One. 2015 Apr 2;10(4):e0121505. doi: 10.1371/journal.pone.0121505 (PMC4383371; doi:10.1371/journal.pone.0121505)
Supplement: S4 Table — (PDF) [file pone.0121505.s012.pdf]

Table S4. Comparative genomic summary of *Frankia* type II polyketide gene cluster biosynthetic proteins and their homologues in pentangular and tetracenomycin training set clusters.

**product**	**species**	**KSα**	**KSβ**	**ACPex, ACPp**	**AroCycN2**	**Cyc2**	**Cyc1**	**ABMh**	**ABMi**	**KR**
frankiamicin	Frankia sp. EAN1pec	FranEAN1<br>_2393	FranEAN1<br>_2394	FranEAN1<br>_2390	FranEAN1<br>_2391	FranEAN1<br>_2392	FranEAN1<br>_2389	FranEAN1<br>_2396	FranEAN1<br>_2397	FranEAN1<br>_2395
frankiamicin	Frankia sp. CcI3	Francci3<br>_2851	Francci3<br>_2850	Francci3<br>_2854	Francci3<br>_2853	Francci3<br>_2852	Francci3<br>_2855	Francci3<br>_2848	Francci3<br>_2847	Francci3<br>_2849
frankiamicin	Frankia alni ACN14a	FRAAL4387	FRAAL4386	FRAAL4390	FRAAL4389	FRAAL4388	FRAAL4392	FRAAL4384	FRAAL4383	FRAAL4385
frankiamicin	Frankia sp. EuI1c	FraEuI1c<br>_4753	FraEuI1c<br>_4752	FraEuI1c<br>_4756	FraEuI1c<br>_4755	FraEuI1c<br>_4754	FraEuI1c<br>_4757	FraEuI1c<br>_4750	FraEuI1c<br>_4749	FraEuI1c<br>_4751
frankiamicin	Frankia sp. EUN1f	FrEUN1f<br>DRAFT_0261	FrEUN1f<br>DRAFT_0262	FrEUN1f<br>DRAFT_0258	FrEUN1f<br>DRAFT_0259	FrEUN1f<br>DRAFT_0260	FrEUN1f<br>DRAFT_0257	FrEUN1f<br>DRAFT_0264	FrEUN1f<br>DRAFT_0265	FrEUN1fDRAFT<br>_0263
pradimicin	Actinomadura hibisca	pdmA	pdmB	pdmC	pdmD	pdmL	pdmK	pdmH	pdmI	pdmG
rubromycin	Streptomyces collinus	rubA	rubB	rubC	rubF<br>(N- terminal)	rubD	rubE	rubH	rubT	rubG
griseorhodin	Streptomyces sp. JP95	grhA	grhB	grhC	grhT<br>(N-terminal)	grhS	grhQ	grhU	grhV	grhO10,<br>grhT (C-terminal)
fredericamycin	Streptomyces griseus	fdmF	fdmG	fdmH	fdmI	fdmE	fdmD	fdmP, fdmJ	fdmQ	fdmO
benastatin	Streptomyces sp. A2991200	benA	benB	benC	benH<br>(N-terminal)	benD	benE	benH	benJ	benL
lysolipin	Streptomyces tendae	llpF	llpE	llpD	llpCI	llpCII	llpCIII	llpOIII	llpOII	llpZI, llpZIII
A-74528	Streptomyces sp. SANK 61196	sanF	sanG	sanH	sanI	sanE	sanD	sanP, sanJ	sanQ	sanO
TLN-05220,<br>TLN-05223	Micromonospora echinospora<br>subsp. challisensis	TLN-ORF18	TLN-ORF19	TLN-ORF20	TLN-ORF21	TLN-ORF17	TLN-ORF16	TLN-ORF23	TLN-ORF24	TLN-ORF22,<br>TLN-ORF14
FD-594	Streptomyces sp. TA-0256	pnxA	pnxB	pnxC, pnxV	pnxD	pnxL	pnxK	pnxH	pnxI	pnxG, pnxW
xantholipin	Streptomyces flavogriseus	xanF	xanE	xanD	xanC1	xanC2	xanC3	xanO7	xanO6	xanZ3, xanZ4
arixanthomycins	Uncultured bacterium	arx16	arx17	arx18	arx19	arx15	arx14	arx22	arx23	arx21, arx27
fasamycin<br>(AZ154)	Uncultured bacterium	ORF23	ORF22	ORF21	ORF20,<br>ORF30	ORF24	ORF19	*ORF15**	*ORF16**	---
lactonamycin	Streptomyces rishiriensis	lct31	lct32	lct24, lct26	lct27	lct30	lct29	*lct33, lct42**	---	---
lactonamycin Z	Streptomyces sanglieri	lcz31	lcz32	lcz24, lcz26	lcz27	lcz30	lcz29	*lcz33**	---	---
tetracenomycin	Streptomyces glaucescens	tcmK	tcmL	tcmM	tcmN<br>(N-terminal)	tcmJ	tcmI	*tcmH**	---	---
elloramycin	Streptomyces olivaceus	elmK	elmL	elmM	elmNI	elmJ	elmI	*elmH**	---	---
